# Post-genotyping optimization of dataset formation could affect genetic diversity parameters: an example of analyses with alpine goat breeds

**DOI:** 10.1186/s12864-021-07802-z

**Published:** 2021-07-17

**Authors:** Neža Pogorevc, Mojca Simčič, Negar Khayatzadeh, Johann Sölkner, Beate Berger, Danijela Bojkovski, Minja Zorc, Peter Dovč, Ivica Medugorac, Simon Horvat

**Affiliations:** 1 Department of Animal science, Biotechnical Faculty, University of Ljubljana, Jamnikarjeva 101, SI-1000 Ljubljana, Slovenia; 2 Division of Livestock Science, Department of Sustainable Agricultural Systems, University of Natural Resources and Life Sciences Vienna, Gregor Mendel Str. 33, A-1180 Vienna, Austria; 3 Department Animal Genetic Resources, AREC Raumberg-Gumpenstein, Institute of Organic Farming and Biodiversity of Farm Animals, 4601 Thalheim b., Wels, Austria; 4 Population Genomics Group, Department of Veterinary Sciences, Faculty of Veterinary Medicine, Ludwig-Maximilians-University Munich, Lena-Christ-Straβe 48, 8215 Martinsried/Planegg, Germany

**Keywords:** Drežnica goat, Slovenian goat breed, Austrian goat breeds, Genetic diversity, Population structure, Admixture, Dataset optimization, Outlier test

## Abstract

**Background:**

Local breeds retained unique genetic variability important for adaptive potential especially in light of challenges related to climate change. Our first objective was to perform, for the first time, a genome-wide diversity characterization using Illumina GoatSNP50 BeadChip of autochthonous Drežnica goat breed from Slovenia, and five and one local breeds from neighboring Austria and Italy, respectively. For optimal conservation and breeding programs of endangered local breeds, it is important to detect past admixture events and strive for preservation of purebred representatives of each breed with low or without admixture. In the second objective, we hence investigated the effect of inclusion or exclusion of outliers from datasets on genetic diversity and population structure parameters.

**Results:**

Distinct genetic origin of the Drežnica goat was demonstrated as having closest nodes to Austrian and Italian breeds. A phylogenetic study of these breeds with other goat breeds having SNP data available in the DRYAD repository positioned them in the alpine, European and global context. Swiss breeds clustered with cosmopolitan alpine breeds and were closer to French and Spanish breeds. On the other hand, the Drežnica goat, Austrian and Italian breeds were closer to Turkish breeds. Datasets where outliers were excluded affected estimates of genetic diversity parameters within the breed and increased the pairwise genetic distances between most of the breeds. Alpine breeds, including Drežnica, Austrian and Italian goats analyzed here, still exhibit relatively high levels of genetic variability, homogeneous genetic structure and strong geographical partitioning.

**Conclusions:**

Genetic diversity analyses revealed that the Slovenian Drežnica goat has a distinct genetic identity and is closely related to the neighboring Austrian and Italian alpine breeds. These results expand our knowledge on phylogeny of goat breeds from easternmost part of the European Alps. The here employed outlier test and datasets optimization approaches provided an objective and statistically powerful tool for removal of admixed outliers. Importance of this test in selecting the representatives of each breed is warranted to obtain more objective diversity parameters and phylogenetic analysis. Such parameters are often the basis of breeding and management programs and are therefore important for preserving genetic variability and uniqueness of local rare breeds.

**Supplementary Information:**

The online version contains supplementary material available at 10.1186/s12864-021-07802-z.

## Background

Local breeds are being recognized as an important way forward to economically, environmentally, and socially sustainable animal production in both developed and developing countries. Likewise, they provide a basis for future studies on diversity, domestication and positional cloning of interesting genes and traits segregating in the breeds. Such rare local breeds demonstrate phenotypes implying that they retained adaptive and selected alleles to thrive in alpine environments with harsh climate conditions that will likely become more widespread as global temperatures continue to rise. Therefore, scientific research on genetic diversity and adaptive traits of rare local breeds is important for conservation and breeding programs.

Taking a global view, mountains present 25% of continental surfaces [1] but more than half of the world’s population relies directly or indirectly on mountain-based resources such as water, energy, minerals, forest and agricultural products [2]. Especially due to emerging climatic changes, mountainous regions already suffer significant impacts on mountain environments, economies and social changes. Local alpine goat breeds, such as those studied in here, stress the conservation value of these breeds that likely harbor adaptive genetic variation, necessary to tackle some of the issues connected with changes in the mountain region environment. Apart from general ecosystem services, these breeds are also of immense importance in cultural heritage and identity [3]. Adaptation of domestic animals in this fast changing environment can be viewed as a general adaptation problem. Local breeds maintained adaptive traits most likely due to low pressure from artificial selection and possibly high natural selection pressure. However, in recent decades a strong focus has been put on high-yielding global breeds, which has led to a decline in the diversity of local adaptive breeds. Decreasing population size and loss of genetic diversity in rare breeds therefore presents a general problem.

**Fig. 1:**
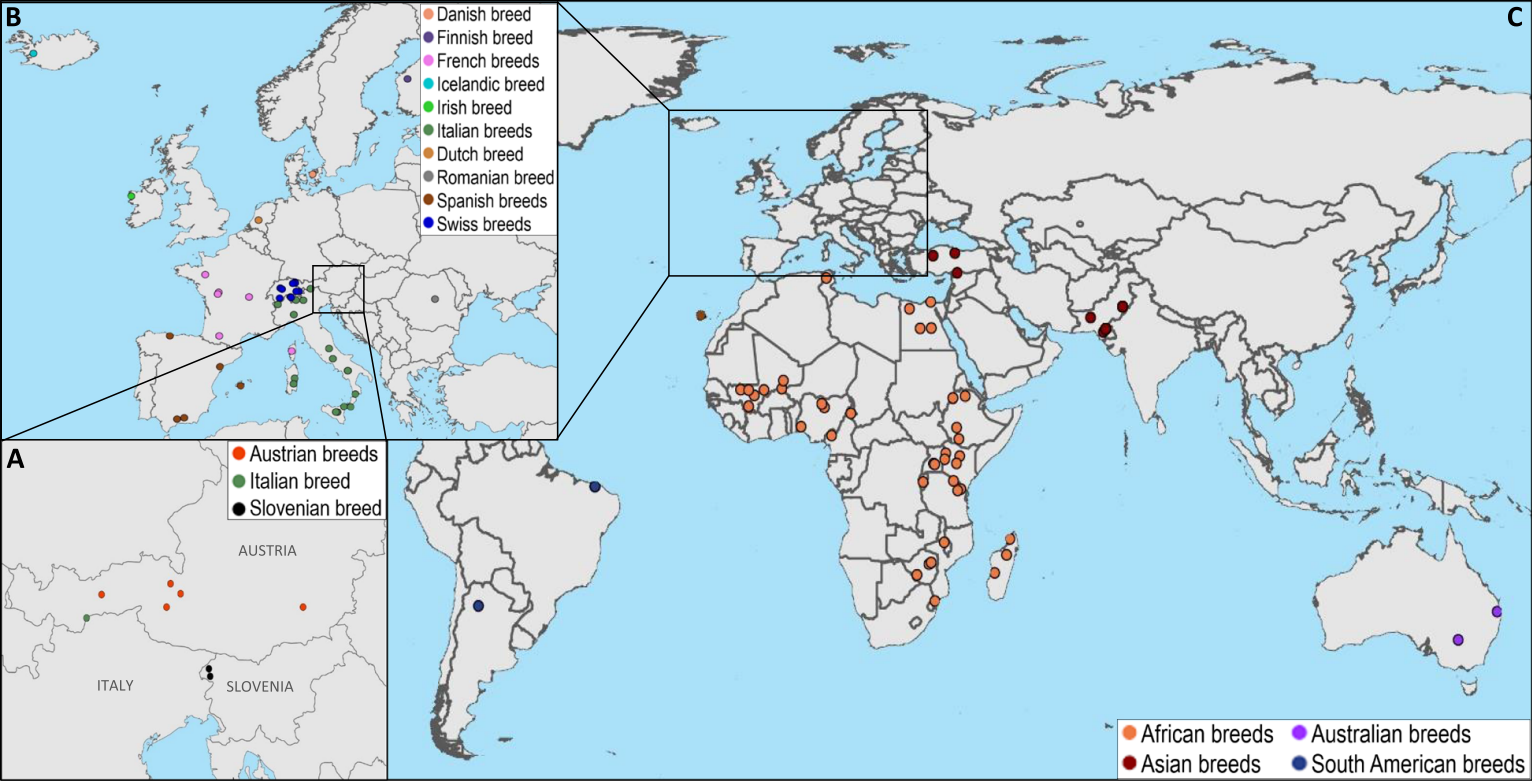
Geographic locations of all goat breeds that were included in our datasets. The SNP genotypes of the Slovenian Drežnica goat (dairy type (upper dot) in the Bovec region; meat type (bottom dot) in the Drežnica region), five Austrian and one Italian goat breeds (**A**) were analyzed together with SNP genotypes of European breeds (**B**) and breeds from other continents (**C**) that were published previously and are available in the DRYAD repository. We created maps with package *rnaturalearth* in the R programming language [4]

To cover local breeds from easternmost part of Alps that have not been investigated in genome-wide diversity studies, we included the only Slovenian local goat breed the Drežnica goat (locally called “drežniška koza”), five goat breeds from Austrian part of Alps (Chamois Colored, Pinzgau, Tauern Pied, Styrian Pied, and Blobe goat) and one goat breed (Passeier goat) from Italian part of Alps (Fig. 1A). These breeds are all from the alpine area of three neighboring countries with long historical ties, in most recent centuries for example under the Habsburg rule between the mid-fourteenth century to 1918. A measure of genome-level variation is an appropriate indicator of how will these breeds respond to the worldwide environmental challenges [5]. For this reason, it is necessary to obtain and compare genome-wide estimates of genetic diversity in local breeds, which are strongly correlated with their long-term response to natural selection in this case [6].

The Drežnica goat originates and primarily still resides in the Slovenian alpine area. The population size of this breed suffered a strong decrease after the Second World War like other local breeds. As a consequence of establishing a herd book and breeding program, the population size has been gradually increasing in the last three decades. However, today the breed is still at a high risk of extinction because of small population size (754 breeding animals in 2019) located in a small area of just 15 km in radius. The Drežnica goat population is divided into two subpopulations according to the production purpose: dairy and meat production type. The dairy subpopulation is mainly widespread around the Bovec region (upper dot in Fig. 1A). Most animals of the dairy type have complete pedigree information beginning in 2000. This subpopulation is still reared according to traditional production system involving indoor rearing during the winter and vertical transhumance during the summer time. Several breeders combine their flocks and use alpine dairy huts for milking and making cheese. Does produce approximately 350 kg of milk over 200 days of lactation with 4.3% fat, 3.4% proteins, and 12% of average dry matter in milk.

The meat type of the Drežnica goat is concentrated in the Drežnica-Kobarid region (bottom dot in Fig. 1A). The production system of this subpopulation is rather unique compared with the intensive modern livestock breeding practices. Animals spend on high mountain pastures about three quarters of a year or more. Goats from several breeders usually comprise a large composite flock (~ 400 animals) that roam and scavenge for their own feed. They rely primarily on the morning dew for a water supply and show exceptional adaptability to varying local weather and seasonal conditions. In late autumn/early winter, the goats are brought back indoors for kidding, and in the early spring, the cycle repeats. Due to implemented production system, the pedigrees are incomplete on the sire side.

Similar goat production systems are present also in neighboring alpine countries of Austria and Italy. The Chamois Colored goat (Gämsfarbige Gebirgsziege Ziege) is a mountain dairy breed originated from Switzerland, developed and distributed through Austria, northern Italy, and France. The breed is predominantly widespread in Tyrol, Vorarlberg, and Upper Austria with a total number of 1806 breeding animals in the year 2016. The Pinzgau goat (Pinzgauer Ziege) is a local dairy mountain breed with thick hair coat that is typically three-colored. In 2016, the total number of registered breeding animals was 963, widespread mainly in Salzburg, Tyrol, and High Tauern National park. The Tauern Pied goat (Tauernschecken Ziege) is a local endangered dairy mountain breed reared in High Tauern around Großglockner mountain. There were 2730 animals registered in the herd book in 2016. The Styrian Pied goat (Steirische Scheckenziege Ziege) is a dairy mountain breed located in the South East of Styria around Graz area. Around 133 breeding animals were registered in the herd book in 2016. The Blobe goat is highly endangered local dual-purpose breed, widespread in the region between the North and South Tyrolean Alpine ridge. The lack of a breeding program for Blobe goat in the past led to a gradual displacement of this breed by Passeier mountain goats by some local farmers due to similar phenotypic characteristics. In the year 2016, only 204 Blobe breeding animals were registered in the herd book. The Passeier goat (Passeier Gebirgsziege or Capra Passiria) is local breed from the Passeier valley or Val Passiria in the autonomous Province of Bolzano (South Tyrol) in northeastern Italy. The breed is also widespread in the neighboring areas of southern Austria, while animals are not registered in the herd book [7, 8].

Recently, the availability of a medium-density single nucleotide polymorphisms (SNP) panel [9] enabled goat genomic studies at a level of resolution that was not possible with previously used markers, such as microsatellites. Several studies have already used this new SNP array tool to analyze the genetic diversity and population structure of local goat breeds or populations within countries in relation to other cosmopolitan breeds, such as in Italy [10], France [11], Switzerland [12], Spain [13], Pakistan [14], China [15], Canada, and Australia [16]. Since the International Goat Genome Consortium (IGGC, http://www.goatgenome.org) was created in 2012, the range of genomic tools and publicly available information for goats has increased immensely [17]. Larger-scale projects within this consortium used this newly developed SNP50K panel to analyze many more goat populations across the world. Topics ranged from examining parentage in worldwide goat breeds [18], post-domestication migration routes [19], homozygosity patterns [18–20], selective sweeps [21] to studies of copy number variation in goat populations [22].

The first objective entailed a genetic diversity study using genome-wide SNP array to investigate whether the Drežnica goat has a distinct genetic identity and, if so, how it relates to the neighboring alpine, especially Austrian breeds, as well as other global breeds. Apart from the study of Luikart et al. [23], who analyzed 8 samples of Drežnica goat in a mtDNA phylogenetic study, Drežnica goat has not been previously included in genomic studies. Likewise, the Austrian local goat breeds included in here also have not been analyzed in a genome-wide study with other European (Fig. 1B) and global (Fig. 1C) breeds. Although the first objective was of more local and practical interest focused on the genetic relationships between the goat breeds from the easternmost part of Alps, we also performed diversity and phylogeny analyses with the wider alpine, European (Fig. 1B) and global goat breeds (Fig. 1C).

In our second objective, we focused on methodology and investigated how different post-genotyping approaches to dataset formation can affect the genetic diversity and population structure parameters. In conservation efforts of rare and especially endangered breeds, it is extremely important to strive for the preservation of purebred individuals and typical representatives of the breed without admixture or with low admixture from other (i.e., cosmopolitan) breeds. Consequently, Ramljak et al. [24] suggested multivariate outlier (*mvOutlier*) analysis to search for admixture signatures. Admixed animals, called outliers, exhibit weaker additive genetic relationships with individuals originating from the same population, stronger genetic relationships with some individuals from other populations, a larger proportion of foreign alleles and an increased number of network connections to individuals of foreign origin. Such animals are not suitable for inclusion in the conservation program, especially admixed males as sire candidates. The exclusion of outliers is not only important for the management of conservation programs but has also a high potential to improve phylogenetic analysis. We here optimized datasets by excluding or including outliers and have shown that this can significantly affect the results of genetic diversity and population structure parameters. We compared all breeds in Alpine datasets by using two post-genotyping optimization approaches. The first one called one-step approach employed removal of closely related animals while the second one (two-step approach) removed admixed outliers first followed by removal of related animals. Our results suggest that the two-step optimization approach can generate datasets that can lead to calculating more objective genetic diversity, population structure, and genetic distance parameters. Finally, we discuss a strategy for conserving and revitalizing small and endangered populations of farm animals, taking into account all the available data.

## Results

### Genetic diversity and the effect of dataset formation

As shown in Table 1, different estimates for genetic diversity parameters within breeds were obtained when analyzing different datasets (*AlpInit*, *Alp1Step*, and *Alp2Step*) that were constructed with or without post-genotyping optimization. The choice of the optimization procedure clearly affected diversity estimates. This was true for nominal values as well as ranges among investigated breeds. For example, among the alpine goat breeds, the Toggenburg breed had the lowest total number of observed alleles (*nA*) (39,223) according to the *AlpInit* and *Alp1Step* datasets. On the other hand, in the *Alp2Step* dataset, the Appenzell goat had the lowest *nA* (35,852). The highest total number of observed alleles in the *Alp1Step* and *Alp2Step* datasets was observed for the Styrian Pied goat, with 65,543 alleles in the one-step dataset optimization and 63,180 in the two-step dataset optimization. Only five of 23 alpine breeds were affected by the one-step procedure, while the two-step procedure affected most breeds (21 out of 23). The Chamois Colored goat from Austria and Peacock goat from Switzerland were the only two breeds that maintained the same sample size after one-step and two-step optimization. For these two breeds, the diversity parameters estimated within the sample remained the same, but the parameters affected by the entire design or by a pair of breeds did not. Consequently, even if identical animals of the Chamois Colored goat from Austria and the Peacock goat were included in all three datasets, the numbers of private (*npA*) and semiprivate (*nrA*) alleles increased in *Alp1Step* and *Alp2Step.* This is due to the exclusion of admixed animals in other breeds from the entire design; i.e., due to admixture, some private alleles became semiprivate or common. The lowest number of private alleles (37, 40 and 43 in *AlpInit, Alp1Step* and *Alp2Step,* respectively) was estimated for the Booted goat, while the highest number of private alleles was estimated for the Drežnica goat (383) based on the *AlpInit* dataset and Styrian Pied goat (326 and 382) according to the *Alp1Step* and *Alp2Step* datasets. The lowest observed heterozygosity (*H*_*O*_) was 0.72 (Appenzell), and the highest was 0.85 (Styrian Pied), based on all three datasets. The average *H*_*O*_ for all alpine breeds in all three datasets was 0.79. Expected heterozygosity (*H*_*E*_) was similar to *H*_*O*_, but the minimum and maximum *H*_*E*_ values were slightly lower than the corresponding *H*_*O*_ values. The range of *H*_*E*_ in *AlpInit* and *Alp1Step* was from 0.70 to 0.84 and in *Alp2Step* was from 0.69 to 0.83. Generally, all populations showed very similar *H*_*E*_ and *H*_*O*_ values regardless of the considered dataset. However, some parameters of allelic diversity, e.g., *npA* and *nrA*, differed substantially (Table 1). Therefore, a comparison of genetic diversity parameters within each breed showed substantial differences when different datasets (*AlpInit*, *Alp1Step*, and *Alp2Step*) were used.

**Table 1:** Genetic diversity parameters of 23 goat populations included in *AlpInit*, *Alp1Step*, and *Alp2Step* datasets

Breed label	N			***nA***			***mA***			***npA (nrA)***			***mpAf***			***Ho***			***He***			***Hdef***		
	***AlpInit***	***Alp1Step***	***Alp2Step***	***AlpInit***	***Alp1Step***	***Alp2Step***	***AlpInit***	***Alp1Step***	***Alp2Step***	***AlpInit***	***Alp1Step***	***Alp2Step***	***AlpInit***	***Alp1Step***	***Alp2Step***	***AlpInit***	***Alp1Step***	***Alp2Step***	***AlpInit***	***Alp1Step***	***Alp2Step***	***AlpInit***	***Alp1Step***	***Alp2Step***
FR_CMA	52	48	33	58450	58037	54400	10.35	10.28	9.64	70 (192)	92 (247)	140 (273)	0.01	0.02	0.02	0.83	0.83	0.83	0.81	0.81	0.80	−0.02	−0.02	− 0.03
IT_VLD	24	24	17	51751	51751	45941	9.17	9.17	8.14	140 (179)	154 (196)	142 (220)	0.04	0.04	0.06	0.76	0.76	0.74	0.78	0.78	0.76	0.03	0.03	0.03
CH_SAA	64	39	38	48967	46885	45864	8.67	8.31	8.12	81 (136)	78 (135)	107 (169)	0.03	0.03	0.04	0.76	0.74	0.76	0.75	0.77	0.75	−0.01	0.04	− 0.01
CH_VAL	43	43	31	44043	44043	42644	7.80	7.80	7.55	68 (128)	71 (143)	84 (171)	0.06	0.06	0.06	0.73	0.73	0.73	0.72	0.72	0.72	−0.01	− 0.01	− 0.01
CH_NVR	42	42	37	55837	55837	54918	9.89	9.89	9.73	90 (180)	100 (193)	130 (249)	0.02	0.02	0.02	0.79	0.79	0.78	0.78	0.78	0.78	0.00	0.00	0.00
CH_TSG	37	37	28	58643	58643	53361	10.39	10.39	9.45	99 (183)	111 (200)	98 (184)	0.03	0.03	0.03	0.81	0.81	0.81	0.80	0.80	0.79	−0.01	− 0.01	− 0.03
CH_TGB	31	31	22	39223	39223	36651	6.95	6.95	6.49	41 (59)	43 (67)	50 (98)	0.04	0.04	0.06	0.73	0.73	0.72	0.71	0.71	0.70	−0.03	− 0.03	− 0.03
CH_APP	29	29	19	39237	39237	35852	6.95	6.95	6.35	45 (77)	47 (87)	43 (113)	0.05	0.05	0.07	0.72	0.72	0.72	0.70	0.70	0.69	−0.03	− 0.03	− 0.04
CH_BOT	23	23	19	42695	42695	41167	7.56	7.56	7.29	37 (75)	40 (79)	43 (98)	0.07	0.07	0.08	0.77	0.77	0.77	0.74	0.74	0.74	−0.04	−0.04	− 0.04
CH_PEA	31	31	31	50873	50873	50873	9.01	9.01	9.01	64 (108)	68 (125)	92 (188)	0.03	0.04	0.04	0.81	0.81	0.81	0.78	0.78	0.78	−0.04	−0.04	−0.04
CH_GST	49	49	38	52107	52107	50723	9.23	9.23	8.99	105 (171)	116 (187)	139 (225)	0.05	0.05	0.05	0.79	0.79	0.79	0.78	0.78	0.77	−0.02	−0.02	−0.02
CH_CHA	123	50	50	60731	56987	54484	10.76	10.10	9.65	70 (154)	71 (120)	67 (135)	0.01	0.01	0.02	0.80	0.80	0.79	0.79	0.79	0.79	−0.01	− 0.01	− 0.01
IT_ORO	23	23	20	46994	46994	44199	8.32	8.32	7.83	85 (137)	91 (150)	105 (175)	0.04	0.04	0.05	0.74	0.74	0.73	0.74	0.74	0.73	0.00	0.00	−0.01
IT_ABL	24	24	20	59640	59640	56520	10.57	10.57	10.01	188 (281)	205 (297)	225 (368)	0.03	0.03	0.03	0.82	0.82	0.83	0.82	0.82	0.81	−0.01	−0.01	− 0.02
IT_CMA	158	50	48	66261	62074	54050	11.74	11.00	9.57	124 (287)	91 (248)	50 (162)	0.01	0.01	0.03	0.81	0.82	0.82	0.82	0.82	0.79	0.01	0.01	−0.03
IT_VLP	24	24	20	60047	60047	57493	10.64	10.64	10.18	157 (293)	171 (315)	191 (363)	0.03	0.03	0.03	0.82	0.82	0.82	0.82	0.82	0.82	0.01	0.01	0.00
IT_PSR	22	22	19	54825	54825	52094	9.71	9.71	9.23	128 (209)	139 (222)	150 (269)	0.03	0.03	0.03	0.81	0.81	0.81	0.80	0.80	0.80	−0.01	−0.01	− 0.01
AT_BLB	34	34	28	60302	60302	58164	10.68	10.68	10.30	155 (308)	170 (332)	189 (377)	0.02	0.02	0.03	0.82	0.82	0.82	0.81	0.81	0.81	−0.01	− 0.01	−0.02
AT_CHA	22	22	22	50588	50588	50588	8.96	8.96	8.96	38 (91)	48 (105)	68 (121)	0.03	0.03	0.03	0.81	0.81	0.81	0.79	0.79	0.79	−0.02	−0.02	−0.02
AT_PNZ	27	27	22	55553	55553	51791	9.84	9.84	9.17	149 (273)	164 (291)	158 (306)	0.03	0.03	0.04	0.79	0.79	0.78	0.79	0.79	0.78	0.00	0.00	0.00
AT_TAP	28	28	24	44555	44555	43262	7.89	7.89	7.66	87 (186)	96 (197)	129 (235)	0.05	0.05	0.06	0.76	0.76	0.77	0.74	0.74	0.74	−0.03	− 0.03	− 0.04
SI_DRZ	133	50	50	64369	58802	55097	11.40	10.42	9.76	383 (520)	290 (439)	301 (482)	0.03	0.03	0.04	0.78	0.79	0.79	0.80	0.79	0.79	0.02	0.01	0.00
AT_STP	32	32	27	65543	65543	63180	11.61	11.61	11.19	301 (495)	326 (545)	382 (607)	0.02	0.02	0.03	0.85	0.85	0.85	0.84	0.84	0.83	−0.01	−0.01	−0.02

*N* sample size

*nA* total number of observed Alleles within subpopulation

*mA* mean number of alleles per block

*npA* number of private Alleles

*nrA* number of alleles present only in two subpopulations (this and anyone other)

*mpAf* mean frequency of private Alleles

*Ho* average observed Heterozygosity

*He* average expected Heterozygosity

*Hdef* Heterozygosity deficiency (He-Ho)/He

The aforementioned differences in genetic diversity parameters within breeds obtained when analyzing different datasets could potentially be due to the effect of differences in the number of genotyped animals being different among breeds and datasets. To control for differences in the number of goats in a dataset, we calculated the mean allelic richness (*mAR*). Among all datasets and breeds, Valdostana goat had the lowest number of animals [18] after the two-step optimization. To obtain differences caused primarily by sampling method and not by minimal sample size, we used the same 17 animals of Valdostana goat in the *AlpInit, Alp1Step* in *Alp2Step* datasets (Table 2). In five alpine breeds affected by the one-step procedure, allelic richness increased. In the two-step procedure, *mAR* decreased (0.2–11.3%) in 14 samples, while increased for 0.3–2.3% in seven samples. In datasets of one- and two-step procedures, the Toggenburg goat had the lowest *mAR*, with 6.17 and 6.15 alleles per locus, while the Styrian Pied goat had the highest *mAR* with 9.96 and 9.94 alleles per locus, respectively. Even though the numbers of animals of the Chamois Colored (Switzerland) and Drežnica goat were the same in the *Alp1Step* and *Alp2Step* datasets, the *mAR* values varied between datasets, because of the different selection of representative animals for both breeds depending on the multivariate outlier analysis. In contrast, the number and selection of animals in the Austrian Chamois Colored and Peacock goats were the same in all datasets- as expected, a follow up analysis yielded the same constant estimates of *mAR.*

**Table 2:** Measures of mean allelic richness (*mAR*) estimated for goat breeds in *AlpInit*, *Alp1Step* and *Alp2Step* standardized on the smallest sample of 17 goats (IT_VLD)

Breed label	N			***mAR***		
	***AlpInit***	***Alp1Step***	***Alp2Step***	***AlpInit***	***Alp1Step***	***Alp2Step***
FR_CMA	52	48	33	8.40	8.42	8.37
IT_VLD	17	17	17	8.14	8.14	8.14
CH_SAA	64	39	38	6.89	7.05	6.96
CH_VAL	43	43	31	6.47	6.47	6.62
CH_NVR	42	42	37	8.07	8.07	8.12
CH_TSG	37	37	28	8.64	8.64	8.35
CH_TGB	31	31	22	6.17	6.17	6.15
CH_APP	29	29	19	6.21	6.21	6.19
CH_BOT	23	23	19	7.05	7.05	7.09
CH_PEA	31	31	31	7.91	7.91	7.91
CH_GST	49	49	38	7.55	7.55	7.62
CH_CHA	123	50	50	7.95	8.14	7.86
IT_ORO	23	23	20	7.63	7.63	7.46
IT_ABL	24	24	20	9.62	9.62	9.55
IT_CMA	158	50	48	8.54	8.91	7.91
IT_VLP	24	24	20	9.70	9.70	9.73
IT_PSR	22	22	19	9.06	9.06	8.95
AT_BLB	34	34	28	9.00	9.00	9.05
AT_CHA	22	22	22	8.39	8.39	8.39
AT_PNZ	27	27	22	8.70	8.70	8.54
AT_TAP	28	28	24	6.99	6.99	7.03
SI_DRZ	133	50	50	8.03	8.23	7.86
AT_STP	32	32	27	9.96	9.96	9.94

**Fig. 2:**
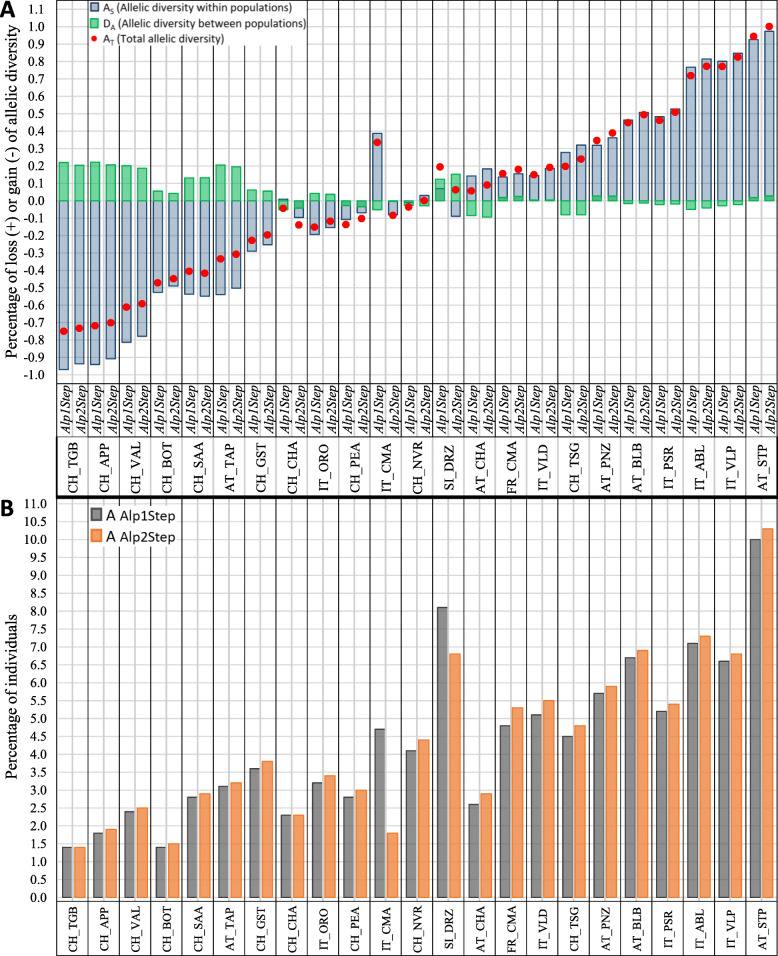
**A** Percentage of loss (+) or gain (−) in allelic diversity within (*A*_*S*_) populations, between populations (*D*_*A*_) and in total (*A*_*T*_) after removing each goat breed from datasets *Alp1Step* and *Alp2Step*; **B** Contributions of individuals from each population in datasets *Alp1Step* and *Alp2Step* to a synthetic pool with the maximal number of alleles (*A*)

Similar to the analyses presented above, differences in genetic diversity parameters results when analyzing differently optimized datasets were also demonstrated by the program *MetaPop*2 (Fig. 2). *MetaPop*2 removed each breed separately from the dataset and estimated the resulting percent change in total (*A*_*T*_), within-population (*A*_*S*_), and between populations (*D*_*A*_) allelic diversity for the remaining animals in the dataset (Fig. 2A). A loss (+) of diversity means a positive contribution of the excluded breed to the allelic diversity, while a gain (−) in diversity after its exclusion implies a negative contribution. As expected, *Alp1Step* and *Alp2Step* showed different results of breed contributions to the total allelic diversity. The largest differences between contributions to *A*_*T*_ in *Alp1Step* compared to *Alp2Step* were found for the Italian Camosciata Alpine (0.419%), Drežnica (0.131%), and Swiss Chamois Colored (0.096%) goats. We noticed that when comparing the *Alp1Step* and *Alp2Step* datasets, the main difference occurred in *A*_*S*_ values, which changed *A*_*T*_ values. As the matter of fact, in the three above mentioned breeds their contributions to *A*_*S*_ changed from positive in *Alp1Step* to negative in *Alp2Step.* In general, breeds with negative input to *A*_*T*_ reduce their negative contribution, comparing *Alp2Step* with *Alp1Step*. On the other side, breeds that had a positive input to *A*_*T*_ in both datasets increased it when *Alp2Step* was used. Changes also occurred in both components of the total allelic diversity, *A*_*S*_ and *D*_*A*_. If we take as an example a removal of Drežnica goat from a dataset, we see that the exclusion of erroneously sampled and admixed animals reduced allelic diversity within the breed (for 0.159%) but increased allelic diversity between (for 0.028%) breeds. As a consequence, the total allelic diversity in *Alp2Step* (0.063%) was lower than in *Alp1Step* (0.131%). We also observed differences when calculating the percentages of individuals of each breed contributing to a pool of 1000 individuals with the maximal total number of alleles (Fig. 2B). Choosing differently optimized datasets affected the results for 21 breeds. Most of them made a larger contribution to a synthetic pool while two breeds (Camosciata Alpine goat from Italy and Drežnica goat) made lower contributions when analyzing *Alp2Step.* Both calculation modes (Fig. 2) in *MetaPop*2 are based on allelic diversity, and depending on the results of either modes, we can make different recommendations for the conservation of the breeds. The results differed after one- and two-step optimization. To avoid misleading conclusions and incorrect decisions in breeding and conservation programs, our analysis emphasizes the importance of using the two-step post-genotyping optimization.

All the results described above illustrate the importance of choosing the representatives of each breed by a two-step approach to obtain more objective values of genetic diversity parameters within each breed. For this reason, we also used a two-step approach (*Alp2Step* dataset) to analyze diversity parameters between the alpine breeds. A follow-up comparison of diversity parameters between breeds showed similar results as the aforementioned analyses from within breed analyses. When using the *Alp2Step* dataset, the Styrian Pied goat showed the highest level of genetic diversity as follows: *nA* (63,180), *mA* (11.19), *npA* (382), *mAR* (9.94), *H*_*O*_ (0.85), and *H*_*E*_ (0.83) (Table 1). In addition to the Styrian Pied goat, we also estimated high diversity parameters in the Blobe, Adamello Blond, Valpassiria, and Drežnica goat breeds. On the other hand, the Appenzell goat had the lowest values, with the exception of *mAR* (6.19), for almost all diversity parameters: *nA* (35,852), *mA* (6.35), *npA* (43), *H*_*O*_ (0.72), and *H*_*E*_ (0.69). A low number of alleles and low heterozygosity-related parameters were also observed for the Toggenburg, Booted and Valais goat breeds. The expected contributions of each breed were consistent with the parameters discussed above (Fig. 2A). The largest loss of the total diversity was observed after removing the Styrian Pied goat (1.001), followed by the Valpassiria (0.826) and Adamello Blond (0.773) goat breeds. Other breeds that contributed positively to *A*_*T*_ were the Passeier, Blobe, Pinzgau, Tessin Grey, Valdostana, Camosciata Alpine (France), Chamois Colored (Austria), Drežnica, and Nera Verzasca goat breeds. In contrast, the largest negative input to total diversity was observed for Toggenburg (− 0.733), Appenzell (− 0.701), and Valais (− 0.592) goat breeds. We observed similar results for these breeds in the case of average allelic diversity within the population, while average allelic diversity between populations produced different results. The Appenzell goat (0.207) made the largest contribution to *D*_*A*_, with the Toggenburg (0.205), Tauern Pied (0.195), Valais (0.187), and Drežnica (0.152) goat breeds ranked after it. The lowest contribution to *D*_*A*_ was made by the Grisons striped goat (− 0.094) together with the Tessin Grey goat (− 0.081). Further, the software provided the optimal number of goats contributed by each population in the *Alp2Step* dataset to create a synthetic population of 1000 animals with the largest total number of alleles (Fig. 2B). After the Styrian Pied goat, larger contributions to the synthetic pool with the maximal number of alleles were observed for the Adamello Blond (7.3), Blobe (6.9), Drežnica (6.8), and Valpassiria (6.8) goat breeds. The percentage of animals that certain breeds contributed to the synthetic pool supported the above listed parameters of allelic diversity.

### Population structure analysis

**Fig. 3:**
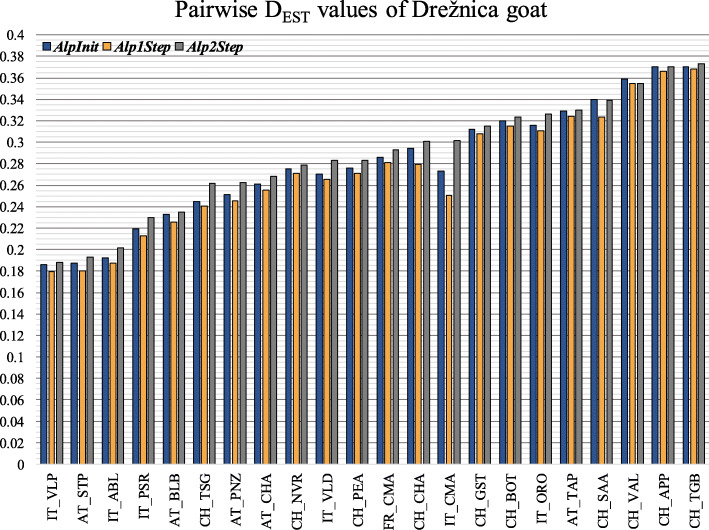
Pairwise *D*_*EST*_ distances between the Drežnica goat and other breeds in alpine datasets. The *D*_*EST*_ distances varied not only between breeds but also among different datasets (*AlpInit*, *Alp1Step*, and *Alp2Step*)

Pairwise population genetic differentiation (Table S3) varied between the one- or two-step optimized alpine datasets. The *G*_*ST*_ values do not necessarily classify populations correctly in terms of their differentiation, especially when *G*_*ST*_ values are high. For this reason, we used *D*_*EST*_ (Table S3) as the chosen population differentiation values because this parameter is independent of heterozygosity. The calculated matrix of differences (*D*_*EST Alp2Step*_ **-** *D*_*EST Alp1Step*_) between *Alp1Step* and *Alp2Step* are shown in Table 3. The differences were on average positive in the case of 15 breeds, meaning that the *D*_*EST*_ values mostly increased when using *Alp2Step* dataset. For example, in *Alp2Step*, the *D*_*EST*_ of the Drežnica goat varied from 0.188 with the Valpassiria goat to 0.373 with the Toggenburg goat (Fig. 3). Considering *Alp1Step*, the pairwise distances of the Drežnica goat from other breeds were lower by 0.014 on average and varied from 0.180 to 0.368. The largest differences were observed for the Italian Camosciata Alpine goat; on average, its *D*_*EST*_ values were higher by 0.040 in *Alp2Step* compared to *Alp1Step.* More specifically, the French Camosciata Alpine goat was the most closely related to the Italian Camosciata Alpine goat, with a pairwise *D*_*EST*_ value of 0.014 in *Alp1Step*, which changed to 0.087 in *Alp2Step*. Similarly, the *D*_*EST*_ value between the Italian Camosciata Alpine goat and the most distantly related goat, the Toggenburg goat, increased from 0.319 in *Alp1Step* to 0.343 in *Alp2Step.* The more objective purebred representatives of a particular breed were selected (i.e., in *Alp2Step* dataset), the larger the *D*_*EST*_ distances between *Alp1Step* and *Alp2Step* became. Breeds with the minimum and maximum values for both parameters had the same position among datasets, but the order of breeds with intermediate *D*_*EST*_ values was not the same between the two optimized datasets. In addition to the results obtained for genetic diversity parameters, the pairwise genetic differentiation analyses also demonstrated substantial variability when different datasets were used.

**Table 3:** Matrix of calculated differences between *D*_*EST*_ values of breeds in *Alp1Step* and *Alp2Step* (*D*_*EST*_ *Alp2Step* - *D*_*EST*_ *Alp1Step*)

	FR_CMA	IT_VLD	CH_SAA	CH_VAL	CH_NVR	CH_TSG	CH_TGB	CH_APP	CH_BOT	CH_PEA	CH_GST	CH_CHA	IT_ORO	IT_ABL	IT_CMA	IT_VLP	IT_PSR	AT_BLB	AT_CHA	AT_PNZ	AT_TAP	SI_DRZ	AT_STP
**FR_CMA**	0																						
**IT_VLD**	0.005	0																					
**CH_SAA**	0.004	0.013	0																				
**CH_VAL**	−0.011	−0.005	−0.008	0																			
**CH_NVR**	−0.005	0.003	0.003	−0.012	0																		
**CH_TSG**	0.008	0.018	0.014	−0.003	0.014	0																	
**CH_TGB**	−0.005	0.001	−0.002	− 0.011	−0.006	0.003	0																
**CH_APP**	−0.004	0.002	−0.001	−0.009	− 0.006	0.002	0.003	0															
**CH_BOT**	−0.004	0.001	0.001	−0.011	− 0.007	0.005	− 0.002	−0.002	0														
**CH_PEA**	−0.001	0.007	0.005	−0.010	− 0.004	0.012	− 0.003	−0.002	− 0.004	0													
**CH_GST**	−0.005	0.001	0.002	−0.014	− 0.007	0.004	− 0.005	−0.005	− 0.007	−0.004	0												
**CH_CHA**	0.018	0.019	0.015	−0.003	0.007	0.020	0.003	0.006	0.003	0.011	0.005	0											
**IT_ORO**	0.003	0.011	0.009	−0.005	0.004	0.017	0.000	0.003	0.003	0.006	0.002	0.016	0										
**IT_ABL**	0.001	0.009	0.012	−0.011	−0.002	0.016	0.046	0.008	0.005	0.005	0.001	0.017	0.009	0									
**IT_CMA**	0.073	0.050	0.038	0.018	0.036	0.052	0.024	0.023	0.025	0.040	0.033	0.033	0.040	0.054	0								
**IT_VLP**	−0.007	0.004	0.005	−0.016	−0.009	0.007	−0.009	−0.008	− 0.009	−0.005	− 0.011	0.010	0.003	−0.003	0.047	0							
**IT_PSR**	0.002	0.011	0.012	−0.008	0.001	0.017	−0.002	− 0.001	−0.001	0.004	−0.003	0.017	0.010	0.005	0.052	0.003	0						
**AT_BLB**	−0.007	0.004	0.003	−0.014	− 0.008	0.007	− 0.007	−0.007	− 0.008	−0.005	− 0.009	0.008	0.003	−0.004	0.043	−0.007	0.000	0					
**AT_CHA**	−0.002	0.007	0.006	−0.011	−0.003	0.009	−0.004	− 0.003	−0.005	0.000	−0.005	0.002	0.006	0.003	0.023	−0.005	0.003	−0.004	0				
**AT_PNZ**	0.003	0.011	0.010	−0.007	0.002	0.016	0.001	−0.001	0.001	0.004	0.000	0.015	0.010	0.008	0.045	0.002	0.010	0.001	0.004	0			
**AT_TAP**	−0.006	0.002	0.001	−0.010	− 0.005	0.008	− 0.007	−0.004	− 0.006	−0.003	− 0.006	0.005	0.002	−0.001	0.025	−0.008	0.000	−0.007	− 0.004	0.002	0		
**SI_DRZ**	0.012	0.018	0.016	0.000	0.008	0.021	0.005	0.004	0.008	0.012	0.007	0.021	0.016	0.014	0.051	0.009	0.017	0.009	0.013	0.017	0.006	0	
**AT_STP**	−0.002	0.007	0.020	−0.012	−0.005	0.013	−0.006	−0.003	− 0.004	0.000	− 0.006	0.014	0.006	0.002	0.051	−0.006	0.003	−0.006	0.000	0.006	0.004	0.013	0
**MIN**	−0.011	− 0.005	−0.008	− 0.016	−0.012	− 0.003	−0.011	− 0.009	−0.011	− 0.010	−0.014	− 0.003	−0.005	− 0.011	0.018	− 0.016	−0.008	− 0.014	−0.011	− 0.007	−0.010	0.000	−0.012
**MAX**	0.073	0.050	0.038	0.018	0.036	0.052	0.046	0.023	0.025	0.040	0.033	0.033	0.040	0.054	0.073	0.047	0.052	0.043	0.023	0.045	0.025	0.051	0.051
**MEAN**	0.003	0.009	0.008	−0.008	0.000	0.013	0.001	0.000	−0.001	0.003	−0.001	0.012	0.008	0.009	0.040	−0.001	0.007	−0.001	0.001	0.007	−0.001	0.014	0.004

**Fig. 4:**
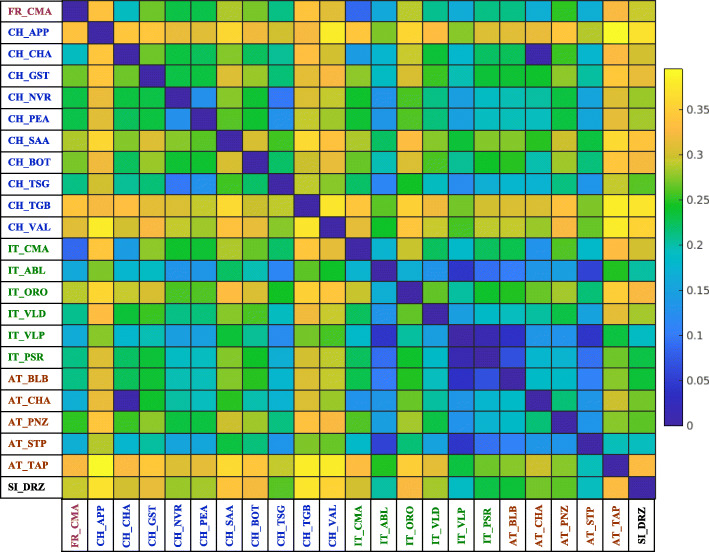
Heatmap representation of pairwise distance values (*D*_*EST*_) among goat breeds in the dataset *Alp2Step*, which was constructed by removing admixed and related animals

To discuss the actual genetic differentiation among populations, we used *D*_*EST*_ values from the *Alp2Step* dataset represented as a heat map in Fig. 4. Five breeds (CH_APP, CH_TGB, CH_VAL, AT_TAP, and SI_DRZ) stood out with higher *D*_*EST*_ values, indicating higher differentiation from other breeds in the alpine set. The Drežnica goat showed higher genetic differences from geographically more distant Swiss (0.262–0.373) and French (0.293) breeds compared to the geographically closer Austrian (0.193–0.330) and Italian (0.188–0.326) breeds. Interestingly, Austrian breeds had lower *D*_*EST*_ values with Italian breeds (0.038–0.350) and higher values with Swiss breeds (0.004–0.395) suggesting that exchanges of sires or the migration historical routes were more frequent between Austria and neighboring Switzerland than between Austria and neighboring Italy. Nevertheless, Austrian (AT_CHA) and Swiss (CH_CHA) alpine global breed had a very short pairwise distance of 0.004, as expected. Comparing the *D*_*EST*_ of breeds within each country, breeds from Switzerland (0.097–0.381) had a higher *D*_*EST*_ among them than breeds from Italy (0.013–0.295) and Austria (0.107–0.298).

**Fig. 5:**
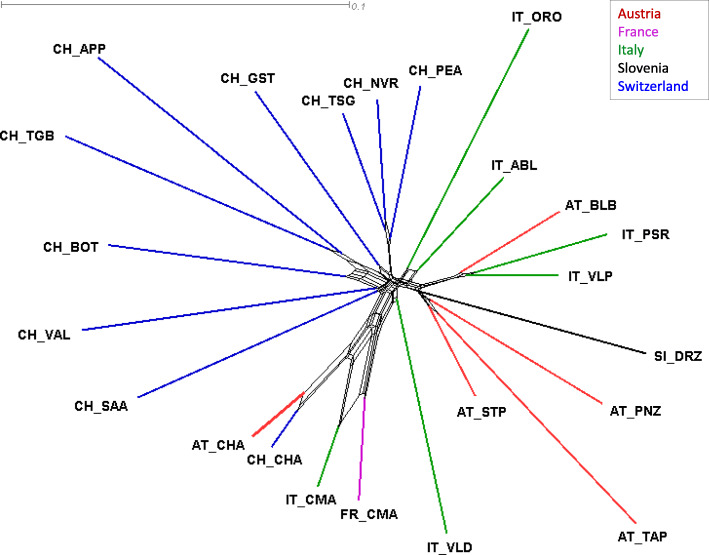
Phylogenetic neighbor net of alpine goat breeds from the two-step (excluding admixed and related animals) optimized dataset constructed with Nei’s *D*_*A*_ distances (scale in the upper left corner) calculated with the 4-SNP blocks

**Fig. 6:**
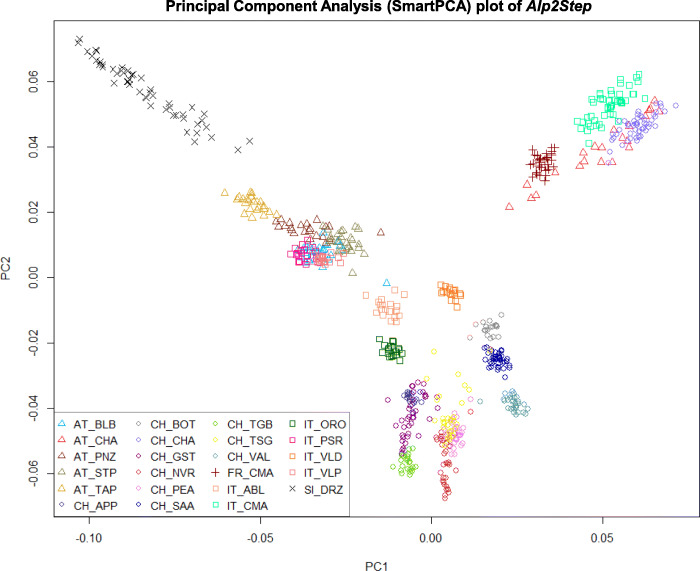
Graph of Principal Component Analysis (PCA) performed with SNP alleles of goat breeds from the *Alp2Step* dataset, where admixed and related animals were excluded

The neighbor-joining trees were plotted with the overall distances between goat breeds in the two-step optimized alpine dataset (Fig. 5), two-step optimized European dataset (Figure S1) and two-step optimized global dataset (Figure S2). Here investigated Drežnica goat as well as Austrian goat breeds were placed closest to geographically neighboring breeds in the phylogenetic neighbor net of alpine goat breeds. The branch of the Drežnica goat breed clearly split from the internal branch shared with other surrounding breeds, confirming that the Drežnica breed is genetically distinct. The Drežnica goat was positioned in the wider cluster of mainly Austrian breeds, which are widespread north to northeast (AT_STP, AT_TAP, and AT_PNZ) or northwest (IT_VLP, IT_PSR, and AT_BLB) of the alpine region, where the Slovenian Drežnica goat resides. Figure 5 also clearly shows very close relationships among the four cosmopolitan breeds (AT_CHA, CH_CHA, IT_CMA, and FR_CMA). The results from the neighbor net graph (Fig. 5) were consistent with the graphs of the principal component analysis with SmartPCA constructed with eigenvectors of *Alp2Step* (Fig. 6) and *Euro2Step* (Figure S3) datasets. Three major groups were separated according to the analysis shown in Fig. 6. All four cosmopolitan breeds (FR_CMA, IT_CMA, CH_CHA, and AT_CHA) formed one cluster, Austrian, Italian and Swiss breeds formed the second cluster, and the Drežnica goat separated out as a third group. The cluster of breeds from Austria, Italy, and Switzerland consisted of three subclusters depending on the country of origin. Austrian breeds were positioned closest to the Drežnica goat, followed by Italian and then Swiss breeds. The above-described genetic relationships of alpine breeds were generally maintained in the PCA plot comparing all European breeds (Figure S3). Breeds from one country broadly grouped together, with the exception of Italian goat breeds that had more dispersed structure and exhibited a separation between breeds originating from the southern or northern part of the country. The most distantly positioned clusters formed Turkish and Icelandic goat breeds, which goes along with their geographic distance.

**Fig. 7:**
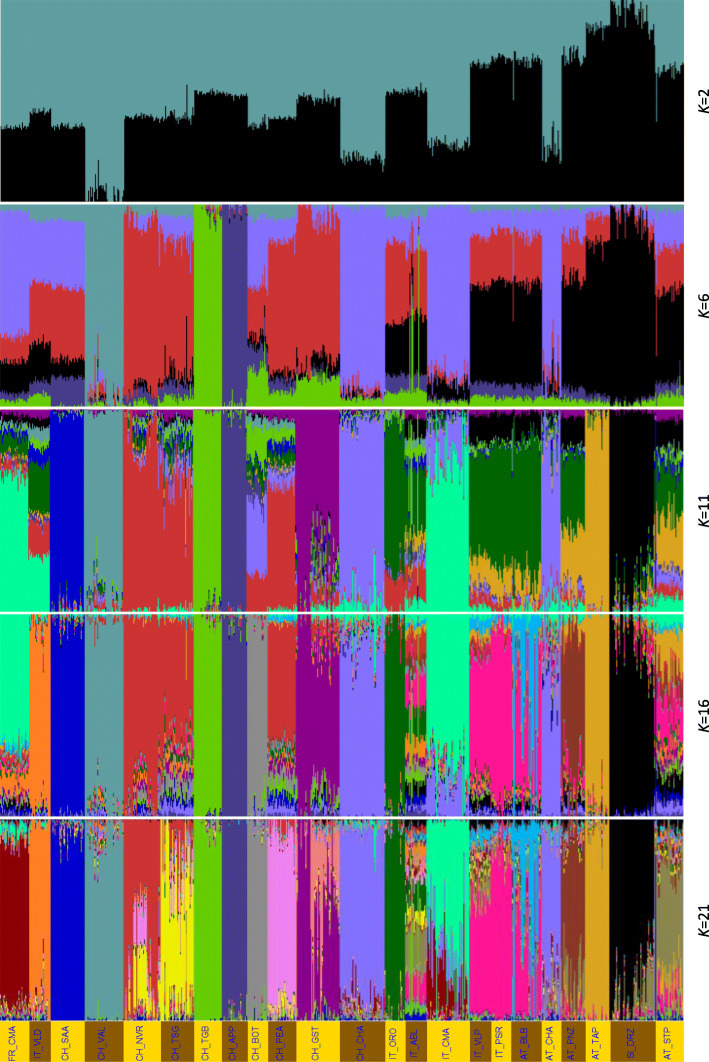
Population structure from *Admixture* analysis for *K* = 2, 6, 11, 16, and 21 of breeds that were optimized with the two-step procedure (*Alp2Step)*, excluding admixed and related animals. The lowest cross-validation error was observed at *K* = 21

The admixture analysis revealed the lowest cross-validation error (CV = 0.607) for the *Alp2Step* set at the optimum number of 21 (*K* = 21) hypothetical populations. The graphical visualization of the results in Fig. 7 shows 11 breeds forming homogeneous clusters, while some individuals or whole populations of other breeds were slightly admixed, causing clusters that are more heterogeneous. In the analysis using the optimum *K* value of 21, populations of Valdostana, Saanen, Valais, Toggenburg, Appenzell, Booted, Orobica, and Tauern Pied goats displayed nearly uniform line blocks. Passeier, Pinzgau, and Drežnica goats displayed primarily homogenous blocks with traces of admixture in certain individuals. On the other hand, Adamello Blond, Blobe, Grisons Striped, and Nera Verzasca goat breeds showed strong admixture signatures with other breeds in the alpine set. With this admixture plot analysis, we obtained more detailed population structures providing further support of previous results observed in PCA and neighbor net graphs.

## Discussion

Genomic characterization is an important step toward implementing efficient breeding and conservation programs for endangered local breeds. Animal genetic diversity is becoming critical for food security and rural development especially in light of changing conditions such as climate change, new or reviving human or animal disease threats, as well as changes in market and societal needs. Local breeds that are well adapted to local and increasingly drier/warmer conditions should play a more prominent role in the livestock production and food security in both developed and developing countries. Better genetic characterization of local goat breeds is essential for more efficient genetic improvement programs targeting adaptive traits and conservation management strategies.

In this study, the genetic diversity analyses of the Slovenian Drežnica goat, five Austrian goat breeds and one from South Tyrol in north-eastern Italy was performed for the first time. A comparison was done with already published alpine goat breeds, most of which are important and rare local breeds. Apart from the importance of these breeds in food production, these breeds also represent cultural heritage of local societies, but they are often threatened by replacement or crossbreeding with more productive cosmopolitan commercial breeds. This process often leads to a significant decrease in population size, which in turn results in inbreeding depression and lower performance, providing additional reasons for their replacement. The worst-case scenario is extinction of local breeds and consequently the loss of key traits for the survival and management of flocks especially in extensive production systems. Traits such as resistance to local diseases, resilience, adaptation to poor forage and water resources, homing and gregarious behavior are crucial in the harsh alpine environment. Many local alpine goat breeds have retained such characteristics, which can help them overcome challenges related to negative effects of climate change. Specifically, the average temperature in the alpine region has risen in recent decades by nearly 2 °C, which is almost twice as large as the average global increase [25]. Genetic characterization studies of rare local breeds such as those studied in here can provide proof of their genetic identity and a basis for maintaining genetic variation, improving their performance and conservation strategies.

Overall, our genetic diversity analysis of goats from the alpine area revealed that they still retained relatively high levels of genetic variability, as was also found in other studies with SNP arrays [8–10, 17]. However, we noted some differences that warrant discussion. Since the goat SNP chip used here was designed based on genomic data from cosmopolitan breeds [9], it could be biased against rare and more diverse local breeds, an issue called ascertainment bias [26]. To mitigate such bias, we used 4-SNP haplotype blocks as marker units rather than single-SNP alleles. Compared with breeds from the neighboring alpine area, the Drežnica goat tends to have a larger total number of observed alleles, mean number of alleles per block and number of private alleles. These results are encouraging, as the breed went through bottlenecks in the past and the population size is low today. The Drežnica goat has the second highest number of population-specific alleles, supporting its distinct genetic identity as a breed. The high number of private alleles could explain its excellent adaptability to the adverse climate/forage conditions in the Alps and indicate that frequent admixture with other alpine breeds was not common. This is likely why the Drežnica goat breed contributes 6.8% to the synthetic pool of alpine breeds with the maximal total number of alleles (Fig. 2B), which is among the top contributions compared to other alpine breeds. The Drežnica goat also contributed positively to total allelic diversity of the *Alp2Step* dataset (Fig. 2A). After removing the Appenzell, Toggenburg, Tauern Pied, Valais, and Drežnica goats individually from *Alp2Step*, the recalculations showed that these breeds accounted for the highest percentage of allelic diversity among the populations. This is in line with the results of the differentiation analysis for these five breeds, whereby they exhibited the highest pairwise distance values from other alpine breeds (Fig. 4). Therefore, based on various genetic diversity analyses, we can conclude that the alpine breeds, including local breeds with small population sizes, generally retained appreciably high levels of genetic variability with a few breeds excelling, such as the Appenzell, Toggenburg, Tauern Pied, Valais, and Drežnica goats, that exhibited the highest percentage of diversity.

The distinct genetic origin of the Drežnica goat was further demonstrated with the neighbor-joining tree (Fig. 5), where the breed formed its own branch. This statement was supported with analysis presented in Fig. 6, where Drežnica goat composed its own cluster that was clearly separated from other alpine breeds. Principal components calculated for all individuals of European breeds (Figure S3A) retained the cluster of Drežnica goat separately and close to other alpine breeds, but surprisingly grouping it together with Landrace goats from Netherlands. This was not so obvious in the neighbor net graph of Alpine (Fig. 5) and European breeds (Figure S1), where the closest node to Drežnica goat branch included three Austrian breeds (Pinzgau, Tauern Pied, and Styrian Pied goats) surrounded by Italian goat breeds. The cluster of Austrian breeds was also the closest on PCA graph of alpine breeds (Fig. 6), followed by a group of Italian breeds and the most distant Swiss breeds. For this reason, we added the third principal component (Figure S3B), which separated the Drežnica goat and Dutch Landrace goat. With the third principal component, Drežnica goat formed a subcluster in the middle of Italian and Austrian breeds. Admixture analysis (Fig. 7**)** revealed that a small group of Drežnica goat animals contains some admixture signatures of the Styrian Pied, a neighboring Austrian goat breed. Despite that, Drežnica goat is one of 11 breeds [9, 10, 17] with the most uniform population ancestral structure. In summary, Drežnica goat stayed genetically very homogeneous, which could be due to factors such as geographic and/or demographic isolation, bottlenecks, genetic drift, and distinctiveness or a combination of these factors.

Generally, the alpine breeds clustered according to the country of origin (Fig. 6) and geographical proximity, which was expected. One exception was a cluster of cosmopolitan breeds from France, Italy, Switzerland, and Austria (FR_CMA, IT_CMA, CH_CHA, and AT_CHA). Moreover, admixture analysis (Fig. 7) revealed that a majority of the genetic ancestry is shared between the pairs IT_CMA-FR_CMA and CH_CHA-AT_CHA suggesting that they are most likely one genetically similar population. This was very likely due to sire semen exchange among these dairy breeds selected for high milk production. Another exception were Italian Passeier and Valpassiria goats positioned in the cluster of Austrian rather than Italian breeds (Fig. 6). The reason for this is probably the geographical location of Passeier and Valpassiria goats on both sides of the state border between North Italy and Southwest Austria. On the neighbor-joining tree (Fig. 5) Italian Passeier goat together with Valpassiria goat clustered together with Austrian Blobe goat. Additionally, the Passeier (this study) and Valpassiria [19] breeds could be regarded as one population based on our admixture analysis (Fig. 7), the later also demonstrating Passeier to Blobe introgression. As these breeds share a relatively small geographic area in the Tyrolean Alps, historical admixture was expected. Similar to the PCA results shown in Burren et al. [12], the Toggenburg and Appenzell goats had the same node, and next to that the Tessin Grey, Nera Verzasca and Peacock goats shared another node (Fig. 5). Tessin Grey, Nera Verzasca, Grisons Striped, and Adamello Blond goats displayed more heterogeneous population structures in our analysis (Fig. 7), compared to studies of Burren et al. [12] and Colli et al. [19].

The positions of populations in our study for the Alpine (Fig. 5), European (Figure S1), and Global datasets (Figure S2) were consistent with those on the PCA, neighbor net, and phylogenetic graphs observed in Colli et al. [19]. When we expanded the alpine neighbor net with other European breeds, the breeds from northern and northwestern Europe separated from the alpine breeds. Swiss breeds together with cosmopolitan alpine breeds were closer to French and Spanish breeds. On the other hand, the Drežnica goat and Austrian as well as Italian breeds were closer to Turkish breeds. A possible explanation of introduction of some Turkish goat stocks to Austria, Slovenia and further west to Italy could be in geographical closeness with the Turkish (Ottoman) empire. This was a state that encompassed in the 600-year period (14-twentieth Century) much of Southeastern Europe, Western Asia, and Northern Africa. In 1520, this empire expanded northwest all the way to what is today essentially the eastern border of Slovenia and Austria. The gene flow from the goat domestication center in Mid-East to Europe are thought to occur via two major routes, Danubian and Mediteranean [27]. This “Turkish” goat migration route via the Balkans during the Ottoman empire could represent another most recent wave of south-Danubian-route introduction of goats to Europe. In the phylogenetic network of the global dataset, the breeds mainly clustered according to the continent. The European dataset ended up grouping with Spanish breeds and breeds from South America followed in the next node from the Spanish breeds. This is in line with historical facts regarding Spanish expansion in the early sixteenth century that also brought Spanish goats to this continent. We can conclude that most alpine goat breeds, especially local breeds with small population sizes, show relatively high homogeneous genetic structure and strong geographical partitioning, whereas larger population-sized cosmopolitan alpine breeds exhibit high admixture and geographic spread.

For the purposes of the second objective, we investigated the effect of inclusion or exclusion of outliers from the breed on the genetic diversity and population structure parameters. A composite test encompassing various metrics was used [24] for the detection of admixed outliers. The detection of purebred animals or excluding outliers is important, especially for analyses of small endangered populations of local breeds that are often in danger of crossing with highly selected commercial cosmopolitan breeds. Likewise, outliers in datasets could affect the estimation of genetic diversity and inference of population structure. Consequently, discrepancies in genetic parameters can be large when datasets with or without outliers are compared. For this reason, we formed different datasets using the multivariate outlier test to exclude outliers. If a repeated test still detected “new” outliers, they were again excluded in the second iteration. Finally, related animals were dropped out of the dataset in the last step. Our follow-up comparative analyses clearly demonstrated that datasets with or without outliers could affect the outcome of analyses. Major effects were observed in parameters such as the total number of observed alleles within a breed, number of private alleles, number of semiprivate alleles and mean number of alleles per block. To illustrate this issue for the case of the Drežnica goat, noticeable differences in results (Tables 1, 2 and Fig. 2) were found when comparing the results of analyses of the *Alp1Step* and *Alp2Step* datasets for all the mentioned parameters. It is important to note that these effects did not arise due to differences in the number of animals per dataset, as each dataset included 50 goats, but rather due to differences in representative animals between the two datasets. Interestingly, we noticed also the effect on the diversity and structure parameters of breeds where no outliers or related animals were detected. For example, the Chamois Colored goat (Austria) and Peacock goat maintained the same number and the same animals across the datasets, but the results for these two breeds were also affected due to different dataset constructions of other breeds. In fast changing environment conditions, the level of within population genetic variation is one of the signals for extinction resistance of the breeds [5]. This emphasizes the need to select the optimal method to form the datasets for evolutionary and conservation genetics analyses. In case of employing the commonly used method in dataset formation without excluding outliers or highly related animals, there is a danger of getting the incorrect results and wrong assessment of actual adaptation capacity for the particular breed.

Changes in estimated genetic diversity parameters were observed in many breeds when different datasets were used. However, it was difficult to ascribe these differences to the compositional differences of the datasets versus the number of animals in the datasets. For this reason, we used the tools that account for differences in sample size among populations (Table 2 and Fig. 2). The relative contributions of the breeds to the total allelic diversity were different when we analyzed the *Alp1Step* or *Alp2Step* datasets. The Drežnica goat, Chamois Colored goat from Switzerland, and Camosciata Alpine goat from Italy displayed the largest changes between the datasets (Fig. 2). The differences in allelic diversity within populations were largely responsible for this change. We can explain this with the fact that excluding admixed outliers decreased the allelic diversity in particular breeds, which consequently also affected their contribution to the global allelic diversity. In the case of the Italian Camosciata Alpine goat, there was a loss of total allelic diversity after removing the breed from *Alp1Step,* but when the program removed the breed from *Alp2Step,* there was a gain in the total allelic diversity. The Camosciata Alpine goat from Italy showed the largest difference in *mAR* as well. In *Alp2Step*, this breed had around 11% lower *mAR* than in *Alp1Step* (Table 2). Together with the Swiss Chamois Colored goat, the Italian Camosciata Alpine goat is a member of the alpine breed cluster (Fig. 6**)** and these two breeds hence share a large proportion of alleles. The removal of admixed animals within individual breed causes a decrease in allelic richness and the average number of private alleles. As shown in Fig. 2A, AT_CHA and FR_CMA positively contributed to the total allelic richness in the *Alp2Step* dataset. In contrast, CH_CHA and IT_CMA were not among the breeds with a positive contribution. The reason for this is most likely that the input of CH_CHA and IT_CMA to total allelic diversity was mostly covered by AT_CHA and FR_CMA, since all four are cosmopolitan breeds that were generated from the same Alpine breed. This was not distinguished in the analysis with the *Alp1Step* dataset. Three breeds with the largest set of animals in *AlpInit* (IT_CMA, CH_CHA, and SI_DRZ) had the highest drop of *mAR* in *Alp2Step*. In their case, a larger number of animals consequently also means a larger number of outliers. This clearly demonstrated, how *mAR* in *Alp1Step* was overestimated, because of outliers that remained in the dataset. After detecting and removing them in *Alp2Step*, we got objective estimates of allelic richness for each breed. Considering both optimized datasets of alpine breeds, we can conclude that the exclusion of admixed and related animals reduces *A*_*S*_ and *A*_*T*_ in breeds with low allelic diversity, but increases *A*_*S*_ and *A*_*T*_ in breeds with high allelic diversity.

Analyses of the population diversity is often the basis for the criteria used in the management of breeding and conservation programs. Besides that, allelic diversity parameters are good indicators of long-term response to natural or artificial selection [6]. Local breeds represent the majority of all datasets in this study and these breeds are particularly under long-term pressure of natural selection in harsh environments due to traditional extensive production systems. Estimates of allelic diversity can be directly linked to rates of adaptation and have a potential to be used as objective conservation criteria of each breed [28]. As we demonstrated, it matters which animals we choose as breed representatives for the diversity analyses to obtain more objective results of genetic diversity and structure to form a basis for informed decisions in the breeding and conservation programs.

Differences in how the datasets were prepared also affected diversity parameters within populations, similar to relationship parameters between breeds. In fact, differences in the number of private alleles between datasets of a particular breed increased or reduced *A*_*T*_ in *Alp2Step*, which contributed to better contrast in clustering and more distinct population differentiation. As an example is analysis in Tables S3 and Fig. 3, exhibiting that *D*_*EST*_ values between the Drežnica goat and other breeds were lower in *Alp1Step* than in *Alp2Step*. This means that removal of admixed Drežnica goats resulted in higher pairwise distances with other populations than removal of closely related Drežnica goats only. Analyses with other breeds showed similar results. However, the largest differences in *D*_*EST*_ values between *Alp1Step* and *Alp2Step* were found for the Italian Camosciata Alpine, Swiss Chamois Colored, and Drežnica goats most likely because these breeds had the largest sample size in the *AlpInit* dataset before optimizing *Alp1Step* and *Alp2Step.* Although the same or similar number of animals in *Alp1Step* and *Alp2Step* represented these breeds*,* the composition of animals in each breed was different between datasets. The highest difference in *D*_*EST*_ values was calculated between IT_CMA and FR_CMA (deviation of 0.073, Table 3). As alluded earlier, semen exchange is common among cosmopolitan breeds, and genetically, these breeds resemble essentially one population (Fig. 7). Regardless, excluding the most admixed animals within each breed sharpened the differences between them, which was clearly demonstrated by the differences in distances based on *Alp1Step* or *Alp2Step* dataset. As expected, eliminating admixed animals from *Alp2Step* on average resulted in an increase in the pairwise distances between most breeds. When we removed significant outliers from each breed in the *Alp2Step* dataset, we obtained more objective distances between breeds in the alpine area.

Our results therefore clearly demonstrate that the procedure used for post-genotyping dataset optimization could have a significant impact on the outcome of genetic diversity and population structure analyses. Non-optimal dataset optimization could lead to erroneous conclusions about genetic diversity, identity and relatedness to other breeds. Choosing an objective method for exclusion of outliers can lead to more accurate and unbiased estimation of allelic diversity. Considering strong correlation of allelic diversity and long-term adaptation to the new optima [1, 2, 29] the improved estimation of allelic diversity could be considered as an important part for improvement of the conservation prioritization. Taking all of our results together, we propose the *mvOutlier* test to be considered as a statistically proven, objective and effective tool for identifying outliers to allow for more reliable genetic parameter estimations, especially in local breeds with small population sizes. Likewise, this tool could be included in conservation and breeding programs to avoid or reduce breeding admixed animals in critically endangered populations. The vectors included in our *mvOutlier* analyses, originally proposed in Ramljak et al. [24], could be improved or replaced by more sophisticated vectors, and any optimization in this sense could be of broad interest.

## Conclusion

Characterization of genetic background and relatedness is an important step in forming the conservation or breeding programs and this process should be carried out very carefully especially for the endangered local breeds. Here we estimated genetic diversity parameters, population structure and possible admixture of Slovenian Drežnica goat, five Austrian and one Italian breed for the first time. Several parameters like high number of population-specific alleles had proven the distinct genetic origin of the Drežnica goat, which was further confirmed with its own branch on the neighbor-joining tree. As expected, the phylogenetic analysis placed Drežnica goat close to Austrian and Italian goat breeds, which follows the geographical positions of breeds and historical ties between these neighboring countries. Commonly, most goat breeds from the alpine area showed relatively homogeneous genetic structure and retained relatively high levels of genetic variability.

Moreover, we demonstrated that optimizing the datasets by excluding or including outliers affected the results of genetic diversity and population structure parameters. We compared two alternative approaches of the post-genotyping optimization for dataset formation. The first one was an approach used commonly in such studies, which is based on removing closely related animals. In the second approach, we added additional step to remove significant admixed outliers followed by removal of related animals. For each animal within the datasets, we estimated various parameters, which composed a matrix used for the follow-up multivariate outlier test procedure and repeated this step until no more outliers remained. We applied these one- and two-step optimization approaches to all breeds in Alpine datasets and used them in comparative analyses. This optimization procedures clearly affected genetic diversity estimates of breeds and pairwise genetic differentiation between them. For this reason, we suggest that the two-step optimization approach in dataset formation can be used in analyses to obtain a more objective genetic diversity, population structure and genetic distance parameters.

## Methods

### Sample and SNP data collection

In 2015–2019, samples of 478 Drežnica goats were collected on family farms, details of breeder names and their addresses are given in Table S1. The procedures for sampling ear tissue of animals for this study followed the protocol detailed in the European Council on Animal Care [30]. About 1 mm ear punch tissue sample was taken using Allflex tissue applicator (Allflex, Somerset West, South Africa). Animals were released after the ear tissue samples were collected. From the collected samples, a dataset of 133 representative animals was prepared and genotyped with the Illumina Goat SNP50 BeadChip [9]. These 133 genotyped animals capture a majority of farms/breeders (*N* = 26), both production types (dairy and meat), both sexes (112 does and 21 bucks), and all main coat color patterns. Furthermore, based on pedigree-based data (Central database for small ruminants in Slovenia), only animals with < 0.25 relationship coefficients were included.

The samples of five goat breeds from Austria and one from Italy (South Tyrol) were provided from two sources: The National Gene Bank of Austria and the Bio Bank Xenogenetik. Genotyping was conducted using Illumina Goat SNP50 BeadChip for all samples including Chamois Colored goat (*n* = 27), Pinzgau goat (*n* = 32), Tauern Pied goat (*n* = 33), Styrian Pied goat (*n* = 33), Blobe goat (*n* = 42), and Passeier goat (*n* = 24).

Additionally, 107 breeds with SNP genotypes already available in the DRYAD repository were used [12, 19, 21, 31, 32]. The geographic area across the Alps was covered by the dataset of 23 breeds (i.e., Alpine dataset) from five countries (Austria, France, Italy, Slovenia, and Switzerland) including data from here collected seven goat breeds that have not been published so far. The other genotypes of goats from France, Italy, and Switzerland were previously published [11, 12, 19]. The Alpine dataset (*AlpInit*; 1075 animals of 23 breeds) was enlarged with the addition of 31 breeds from the rest of Europe and called the European dataset (*EuroInit*; 1920 animals of 54 breeds). Furthermore, the European dataset became a part of a global dataset (*GlobInit*; 3943 animals of 114 breeds), where all goat breeds of the world available in the open source repository were included.

### Formation of optimized datasets by multivariate outlier analysis

**Fig. 8:**
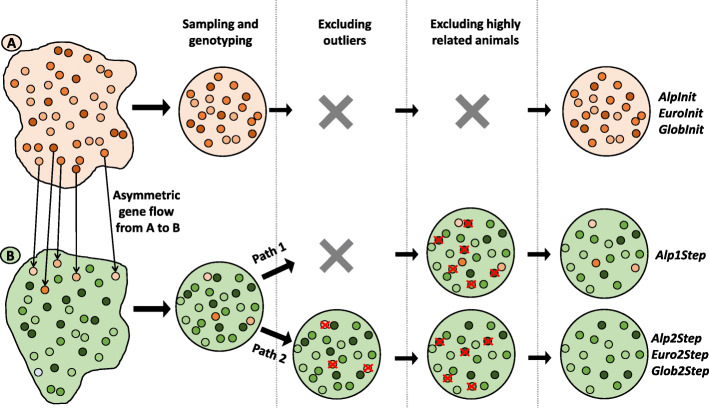
The graphical explanation of the method for constructing the datasets. For example, two resource populations, **A** and **B**, were sampled to investigate genetic diversity. Resource population **A** was used to upgrade population **B**; i.e. there is asymmetric gene flow from **A** to **B**. To create a diversity sample consisting of random animals with relationships not stronger than the average in the resource population, researchers use written and verbal information. After sampling and genome-wide genotyping, some closely related samples could and should be excluded from further analyses requiring unrelated individuals (population structure and phylogenetic analyses). Successive exclusion of one from the most closely related pair of animals is commonly used approach and resulted in a one-step improved diversity sample (*Alp1Step*), as illustrated in the first path of population **B**. Foreign or admixed individuals are prone to weaker relationships within the sample, and consequently, after the one-step procedure, they remain in the improved diversity sample. A preceding outlier test (path 2 for **B**) will decrease the inflated diversity within and increase the diversity between two-step improved diversity samples (*Alp2Step*, *Euro2Step* and *Glob2Step*)

Two alternative post-genotyping approaches were used to create optimized diversity dataset consisting of random animals with relationships not stronger than the average in the resource population (Fig. 8):

(i) The commonly used approach in diversity studies uses genome-wide genotypes to infer additive genetic relationships and successively excludes closely related animals. We applied this one-step approach to 23 goat breeds within the initial Alpine dataset (*AlpInit*) to create the *Alp1Step* dataset (path 1 on Fig. 8).
(ii) We proposed and used herein a two-step approach that first excludes outliers (erroneously sampled and/or admixed animals) by multivariate outlier analysis and then successively excludes closely related animals to further reduce relatedness. We applied this two-step approach to 23 goat breeds of the *AlpInit* dataset to create the *Alp2Step* dataset. The same two-step approach was applied to every breed in *EuroInit* and *GlobInit* to obtain the optimized diversity datasets *Euro2Step* and *Glob2Step* (path 2 on Fig. 8)*.*

The descriptions of breeds, the number of samples and their origins are shown in Table S2. When the initial datasets (*AlpInit, EuroInit*, and *GlobInit*) were constructed, all breeds were included under three conditions: the number of successfully genotyped animals within the breed had to be higher than 13, the animals were not crossbred, and the samples of the breed originated from the country of origin for that particular breed.

The multivariate outlier test (*mvOutlier* [33];) was used for dataset formation adapted from the study of Ramljak et al. [24]. For each animal within the *AlpInit*, *EuroInit*, and *GlobInit* datasets, we estimated various parameters, which composed a matrix used for the follow-up multivariate outlier test procedure. In the first step, we removed significant outliers for each breed in the *AlpInit*, *EuroInit*, and *GlobInit* datasets and repeated this step until no more outliers remained. After the outlier test was completed, closely related animals were excluded based on the unified additive relationships (UAR) matrix [34]. In the second step, we detected and removed animals closely related to one or more animals of the same breed. We iteratively re-estimated the UAR matrix and excluded closely related animals until the maximal relationship stayed below the chosen threshold (UAR > 0.25). These two-step procedures formed datasets *Alp2Step*, *Euro2Step* and *Glob2Step* (Table 4). We used the two-step datasets for analyses that required population-representative and unrelated individuals (e.g., for diversity, phylogenetic and population structure analyses). We compared diversity parameters based on the *Alp1Step* and *Alp2Step* (path 1 and path 2 on Fig. 8) datasets to assess the possible bias introduced by the commonly used one-step approach to optimize the diversity samples.

**Table 4:** Description of the datasets that were formed with different approaches

Dataset	Code	Number of animals	Exclusion of outliers (multivariate test)	Exclusion of related animals (UAR matrix)
Initial Alpine dataset	*AlpInit*	1075		
One-step optimized Alpine dataset	*Alp1Step*	782		✔
Two-step optimized Alpine dataset	*Alp2Step*	663	✔	✔
Initial European dataset	*EuroInit*	1920		
Two-step optimized European dataset	*Euro2Step*	1293	✔	✔
Initial Global dataset	*GlobInit*	3943		
Two-step optimized Global dataset	*Glob2Step*	2132	✔	✔

### SNP and haplotype data processing

DNA was extracted using the Qiagen DNeasy® Blood and Tissue Kit following the manufacturers’ protocols. All genotypes of goat breeds listed in Table S2 in our study or other studies were obtained using the same version of Illumina Caprine 50 K SNP BeadChip (http://www.illumina.com).

The quality control procedures excluded SNPs with genotyping errors (based on available genotypes of relatives), unknown chromosomal positions according to the *Capra hircus* genome assembly ARS1 (https://www.ncbi.nlm.nih.gov/assembly/GCF_001704415.1; autosomal SNPs only), a call rate < 95%, a minor allele frequency < 0.025. Finally, 48,246 autosomal SNPs in the *AlpInit* dataset, 48,288 in the *EuroInit* dataset and 48,297 in the *GlobInit* dataset were considered for the analyses, with an average marker density of 60.5 kb.

Haplotypes were inferred and missing genotypes were imputed using hidden Markov models with the software package *Beagle* version 4.1 [35]. Genome-wide relationships among all individuals were estimated as UARs among animals, which are based on identity by descent (IBD) between corresponding gametes [32, 36]. We used the UAR matrix to reduce familial structures within the populations through the exclusion of closely related animals in the process of optimized datasets formation (see previous section).

### Haplotype diversity and genetic variability analyses

To reduce the ascertainment bias of the Illumina Caprine 50 K BeadChip, we used short haplotypes instead of single SNPs as demonstrated in our previous study [37]. We divided the genome into non-overlapping blocks of four SNP genotypes (4­SNP block) for further analyses. The selected SNP blocks showed distances between neighboring SNPs of less than 50 kb (maximal length of each < 150 kb). As detailed in the SNP quality control procedures above, the number of informative SNPs differed slightly from dataset to dataset. Consequently, the number of SNP blocks used differed minimally: 5645 for *AlpInit*, *Alp1Step* and *Alp2Step*, 5652 blocks for *EuroInit* and *Euro2Step* and 5658 blocks for *GlobInit* and *Glob2Step*.

Distinct haplotypes across and within breeds for each 4­SNP block were counted and the following parameters of allelic diversity were estimated: the total number of observed alleles (*nA*), mean number of alleles per block (*mA*), number of private alleles (*npA*, i.e., alleles observed in only one subpopulation), and number of semiprivate alleles (*nrA*, i.e., alleles observed in only two subpopulations). To reduce the effect of sample size on the number of distinct haplotypes, we estimated allelic richness (*AR*) [38]. We also determined the observed (*H*_*O*_) and expected (*H*_*E*_) heterozygosity [39] and *F* statistics for each block [29]. Population differentiation was estimated with *D*_*EST*_, which is independent of heterozygosity [40]. We used the datasets *Alp1Step* and *Alp2Step* in the program *MetaPop*2 [4] to analyze the contribution of each breed to the total allelic diversity of alpine goat breeds with two different approaches. First, the contribution of each breed was estimated by disregarding that breed and re-estimating the within-population (*A*_*S*_), among-population (*D*_*A*_) and total (*A*_*T*_) allelic diversity of the remaining *Alp1Step* or *Alp2Step* dataset. The second approach involved choosing the optimal number of individuals from each of 23 breeds to create a synthetic population of 1000 individuals with the largest total number of alleles (*A*).

### Population structure analyses

Genetic relationships between the individuals and breeds were revealed with supervised and unsupervised approaches. For supervised clustering, we used 4-SNP blocks, while for unsupervised clustering we used single-SNP alleles. First, we used the final two-step datasets (*Alp2Step*, *Euro2Step* and *Glob2Step;* Table 4) to reconstruct the phylogeny of the breeds based on supervised methods. Nei’s distances (*D*_*Nei*_) [39] were calculated with the 4-SNP blocks, and later, we used the *D*_*Nei*_ distances for the construction of a phylogenetic neighbor net with the program *SplitsTree*4 [41]. Further, to determine the population structure, we used single-SNP alleles and analyzed them with SmartPCA tool [42] from package EIGENSOFT version 7.2.1 [43]. Graphical representations of the outputted eigenvectors were made using the R programming language [44]. These analyses were carried out in a two-step optimized alpine dataset of 663 animals (*Alp2Step*), and in a two-step optimized European dataset of 1293 animals (*Euro2Step*). In addition to that, we also investigated population structure based on the 48,288 autosomal SNPs in the *Alp2Step* dataset by the *Admixture* program [45]. To derive the most likely number of populations (*K*), the 20-fold cross-validation error was estimated for *K* = 2 to *K* = 25. The clustering with the lowest cross-validation error [45] suggested the *K* value of 21 as the most appropriate in our case. The *Admixture* results were plotted using the R programming language [44].

## Supplementary Information

**Additional file 1: Table S1.** The samples of 478 Drežnica goats were collected at 26 Slovenian breeders/farms listed below together with their addresses.

**Additional file 2: Table S2.** Description of global goat breeds with SNP genotypes available in the DRYAD repository.

**Additional file 3: Table S3.** Pairwise *D*_*EST*_ distances among all breed pairs from *Alp1Step* (above) and *Alp2Step* (below) datasets.

**Additional file 4: Figure S1.** Phylogenetic neighbor net of European goat breeds from the two-step (excluding admixed and related animals) optimized dataset constructed with Nei’s *D*_*A*_ distances (scale in the upper left corner) calculated with the 4-SNP blocks.

**Additional file 5: Figure S2.** Phylogenetic neighbor net of global goat breeds from the two-step (excluding admixed and related animals) optimized dataset constructed with Nei’s *D*_*A*_ distances (scale in the upper left corner) calculated with the 4-SNP blocks.

**Additional file 6: Figure S3.** Graph of Principal Component Analysis (PCA) performed with SNP alleles of goat breeds from the *Euro2Step* dataset, where admixed and related animals were excluded. Besides first and second principal components (**A**), the third principal component was also analyzed (**B**).

## Data Availability

The authors declare that most data supporting the findings of this study are available within the article and its supplementary information files. The raw SNP genotype data have been deposited in the NCBI database GEO with accession number GSE176157 and the following web link: https://www.ncbi.nlm.nih.gov/geo/query/acc.cgi?acc=GSE176157.

## Abbreviations

A: Total number of alleles
AlpInit: Initial Alpine dataset
Alp1Step: One-step optimized Alpine dataset
Alp2Step: Two-step optimized Alpine dataset
AR: Allelic richness
A_S_: Within-population allelic diversity
A_T_: Total allelic diversity
CV: Cross-validation error
D_A_: Among-population allelic diversity
D_EST_: Population differentiation values
D_Nei_: Nei’s distances
EuroInit: Initial European dataset
Euro2Step: Two-step optimized European dataset
GlobInit: Initial Global dataset
Glob2Step: Two-step optimized Global dataset
H_O_: Observed heterozygosity
H_E_: Expected heterozygosity
IBD: Identity by descent
K: Number of populations
kb: Kilo base pair
mA: Mean number of alleles per block
mAR: Mean allelic richness
nA: Total number of observed alleles
npA: Number of private alleles
nrA: Number of semiprivate alleles
PCA: Principal component analysis
SNP: Single nucleotide polymorphisms
UAR: Unified additive relationships

## References

[1] Kapos V, Rhind J, Edwards M, Price M, Ravilious C, Butt N. Forests in sustainable mountain development: a state of knowledge report for 2000. Task Force For Sustain Mt Dev. 2000;5:4–19.

[2] Beniston M (2003). Climatic change in mountain regions: a review of possible impacts. Clim Chang.

[3] Marsoner T, Egarter Vigl L, Manck F, Jaritz G, Tappeiner U, Tasser E (2018). Indigenous livestock breeds as indicators for cultural ecosystem services: a spatial analysis within the Alpine space. Ecol Indic.

[4] López-Cortegano E, Pérez-Figueroa A, Caballero A (2019). metapop2: re-implementation of software for the analysis and management of subdivided populations using gene and allelic diversity. Mol Ecol Resour.

[5] Ørsted M, Hoffmann AA, Sverrisdóttir E, Nielsen KL, Kristensen TN (2019). Genomic variation predicts adaptive evolutionary responses better than population bottleneck history. PLoS Genet.

[6] Caballero A, García-Dorado A (2013). Allelic diversity and its implications for the rate of adaptation. Genetics..

[7] ÖBSZ (2008). Österreichischer Bundesverband für Schafe und Ziegen.

[8] ÖNGENE (1982). Österreichische Nationalvereinigung für Genreserven landwirtschaftlicher NutztiereNo Title.

[9] Tosser-Klopp G, Bardou P, Bouchez O, Cabau C, Crooijmans R, Dong Y, et al. Design and characterization of a 52K SNP chip for goats. PLoS One. 2014;9(1):e0152632. 10.1371/journal.pone.0152632. 10.1371/journal.pone.0086227 PMC3899236 24465974

[10] Nicoloso L, Bomba L, Colli L, Negrini R, Milanesi M, Mazza R (2015). Genetic diversity of Italian goat breeds assessed with a medium-density SNP chip. Genet Sel Evol.

[11] Oget C, Servin B, Palhière I (2019). Genetic diversity analysis of French goat populations reveals selective sweeps involved in their differentiation. Anim Genet.

[12] Burren A, Neuditschko M, Signer-Hasler H, Frischknecht M, Reber I, Menzi F, Drögemüller C, Flury C (2016). Genetic diversity analyses reveal first insights into breed-specific selection signatures within Swiss goat breeds. Anim Genet.

[13] Manunza A, Noce A, Serradilla JM, Goyache F, Martínez A, Capote J (2016). A genome-wide perspective about the diversity and demographic history of seven Spanish goat breeds. Genet Sel Evol.

[14] Kumar C, Song S, Dewani P, Kumar M, Parkash O, Ma Y, Malhi KK, Yang N, Mwacharo JM, He X, Jiang L (2018). Population structure, genetic diversity and selection signatures within seven indigenous Pakistani goat populations. Anim Genet.

[15] Berihulay H, Li Y, Liu X, Gebreselassie G, Islam R, Liu W, Jiang L, Ma Y (2019). Genetic diversity and population structure in multiple Chinese goat populations using a SNP panel. Anim Genet.

[16] Brito LF, Kijas JW, Ventura RV, Sargolzaei M, Porto-Neto LR, Cánovas A (2017). Genetic diversity and signatures of selection in various goat breeds revealed by genome-wide SNP markers. BMC Genomics.

[17] Stella A, Nicolazzi EL, Van Tassell CP, Rothschild MF, Colli L, Rosen BD (2018). AdaptMap: exploring goat diversity and adaptation. Genet Sel Evol.

[18] Talenti A, Palhière I, Tortereau F, Pagnacco G, Stella A, Nicolazzi EL (2018). Functional SNP panel for parentage assessment and assignment in worldwide goat breeds. Genet Sel Evol.

[19] Colli L, Milanesi M, Talenti A, Bertolini F, Chen M, Crisà A (2018). Genome-wide SNP profiling of worldwide goat populations reveals strong partitioning of diversity and highlights post-domestication migration routes. Genet Sel Evol.

[20] Cardoso TF, Amills M, Bertolini F, Rothschild M, Marras G, Boink G (2018). Patterns of homozygosity in insular and continental goat breeds. Genet Sel Evol.

[21] Bertolini F, Servin B, Talenti A, Rochat E, Kim ES, Oget C (2018). Signatures of selection and environmental adaptation across the goat genome post-domestication 06 biological sciences 0604 genetics. Genet Sel Evol.

[22] Liu M, Zhou Y, Rosen BD, Van Tassell CP, Stella A, Tosser-Klopp G (2019). Diversity of copy number variation in the worldwide goat population. Heredity (Edinb).

[23] Luikart G, Gielly L, Excoffier L, Vigne JD, Bouvet J, Taberlet P (2001). Multiple maternal origins and weak phylogeographic structure in domestic goats. Proc Natl Acad Sci U S A.

[24] Ramljak J, Bunevski G, Bytyqi H, Marković B, Brka M, Ivanković A, Kume K, Stojanović S, Nikolov V, Simčič M, Sölkner J, Kunz E, Rothammer S, Seichter D, Grünenfelder HP, Broxham ET, Kugler W, Medugorac I (2018). Conservation of a domestic metapopulation structured into related and partly admixed strains. Mol Ecol.

[25] Auer I, Böhm R, Jurkovic A, Lipa W, Orlik A, Potzmann R, Schöner W, Ungersböck M, Matulla C, Briffa K, Jones P, Efthymiadis D, Brunetti M, Nanni T, Maugeri M, Mercalli L, Mestre O, Moisselin JM, Begert M, Müller-Westermeier G, Kveton V, Bochnicek O, Stastny P, Lapin M, Szalai S, Szentimrey T, Cegnar T, Dolinar M, Gajic-Capka M, Zaninovic K, Majstorovic Z, Nieplova E (2007). HISTALP—historical instrumental climatological surface time series of the greater Alpine region. Int J Climatol.

[26] Malomane DK, Reimer C, Weigend S, Weigend A, Sharifi AR, Simianer H (2018). Efficiency of different strategies to mitigate ascertainment bias when using SNP panels in diversity studies. BMC Genomics.

[27] Amills M, Capote J, Tosser-Klopp G (2017). Goat domestication and breeding: a jigsaw of historical, biological and molecular data with missing pieces. Anim Genet.

[28] Vilas A, Pérez-Figueroa A, Quesada H, Caballero A (2015). Allelic diversity for neutral markers retains a higher adaptive potential for quantitative traits than expected heterozygosity. Mol Ecol.

[29] Weir BS, Cockerham CC (1984). Estimating F -statistics for the analysis of population structure. Evolution (N Y).

[30] Europe C of. European convention for the protection of vertebrate animals used for experimental and other scientific purposes. Cets. 1991;(170):123 Available from: http://www.coe.int/en/web/conventions/full-list/-/conventions/treaty/123.

[31] Bertolini F, Cardoso TF, Marras G, Nicolazzi EL, Rothschild MF, Amills M (2018). Genome-wide patterns of homozygosity provide clues about the population history and adaptation of goats. Genet Sel Evol.

[32] Visser C, Lashmar SF, Van Marle-Köster E, Poli MA, Allain D (2016). Genetic diversity and population structure in south African, French and Argentinian angora goats from genome-wide SNP data. PLoS One.

[33] Filzmoser P, Garrett RG, Reimann C (2005). Multivariate outlier detection in exploration geochemistry. Comput Geosci.

[34] Yang J, Benyamin B, McEvoy BP, Gordon S, Henders AK, Nyholt DR (2010). Common SNPs explain a large proportion of the heritability for human height. Nat Genet.

[35] Browning SR, Browning BL (2007). Rapid and accurate haplotype phasing and missing-data inference for whole-genome association studies by use of localized haplotype clustering. Am J Hum Genet.

[36] Powell JE, Visscher PM, Goddard ME (2010). Reconciling the analysis of IBD and IBS in complex trait studies. Nat Rev Genet.

[37] Simčič M, Smetko A, Sölkner J, Seichter D, Gorjanc G, Kompan D (2015). Recovery of native genetic background in admixed populations using haplotypes, phenotypes, and pedigree information - using cika cattle as a case breed. PLoS One.

[38] El Mousadik A, Petit RJ (1996). High level of genetic differentiation for allelic richness among populations of the argan tree [Argania spinosa (L.) Skeels] endemic to Morocco. Theor Appl Genet.

[39] CLEGG MT (1987). Molecular evolution: molecular evolutionary genetics. Science.

[40] Jost L (2008). GST and its relatives do not measure differentiation. Mol Ecol.

[41] Huson DH, Bryant D (2006). Application of phylogenetic networks in evolutionary studies. Mol Biol Evol.

[42] Price AL, Patterson NJ, Plenge RM, Weinblatt ME, Shadick NA, Reich D. Principal components analysis corrects for stratification in genome-wide association studies. Nat Genet. 2006;38(8):904–9. 10.1038/ng1847 16862161

[43] Patterson N, Price AL, Reich D. Population structure and eigenanalysis. PLoS Genet. 2006;2(12):e190. 10.1371/journal.pgen.0020190. 10.1371/journal.pgen.0020190 PMC1713260 17194218

[44] R Core Team (2018). R: A language and environment for statistical computing.

[45] Alexander DH, Novembre J, Lange K (2009). Fast model-based estimation of ancestry in unrelated individuals. Genome Res.

