# Supplementary figures and images for: Post-genotyping optimization of dataset formation could affect genetic diversity parameters: an example of analyses with alpine goat breeds
Source: BMC Genomics. 2021 Jul 17;22:546. doi: 10.1186/s12864-021-07802-z (PMC8285797; doi:10.1186/s12864-021-07802-z)

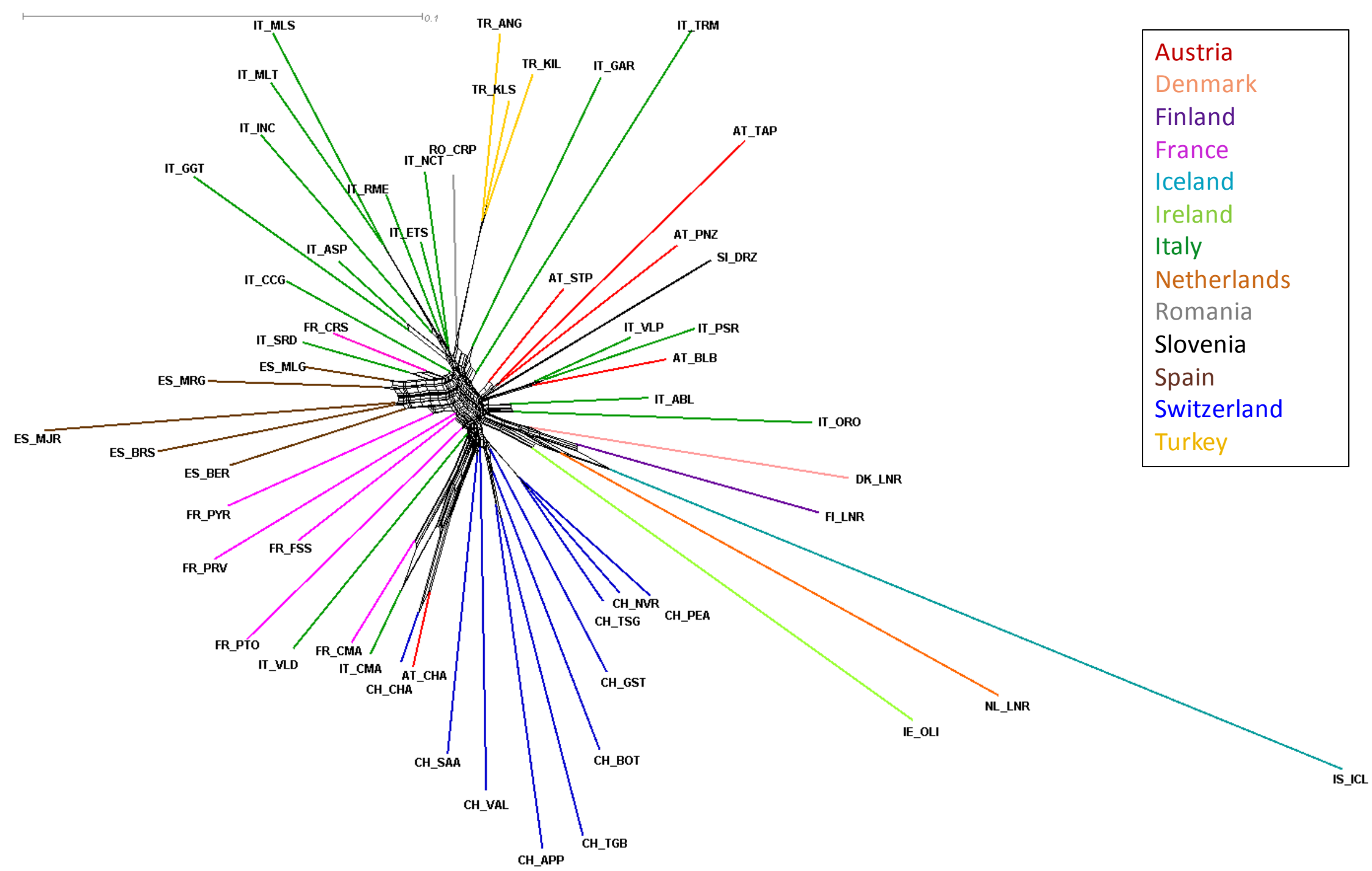

Supplement: Supplementary file 4 — Additional file 4: Figure S1. Phylogenetic neighbor net of European goat breeds from the two-step (excluding admixed and related animals) optimized dataset constructed with Nei’s DA distances (scale in the upper left corner) calculated with the 4-SNP blocks. [file 12864_2021_7802_MOESM4_ESM.pdf]

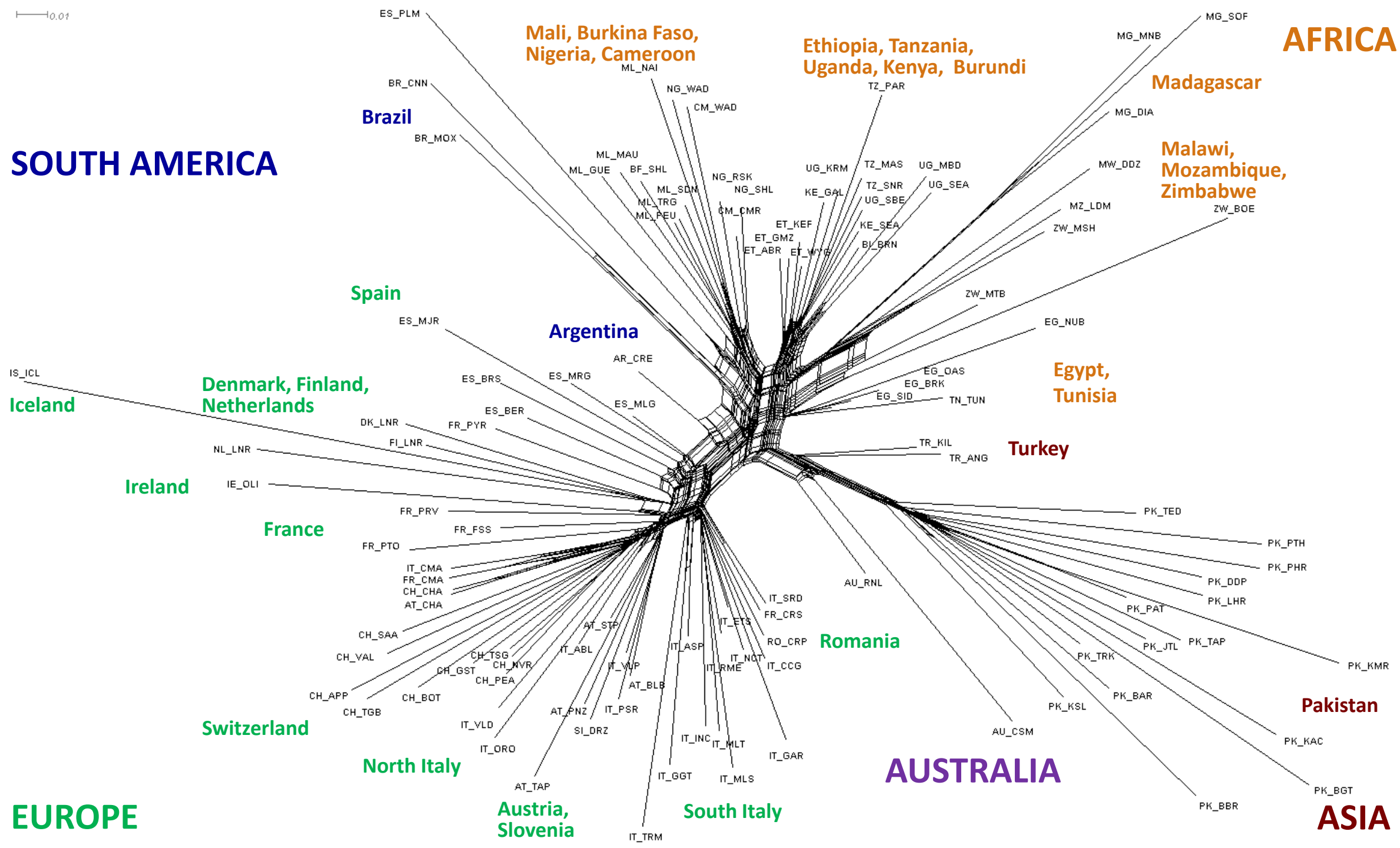

Supplement: Supplementary file 5 — Additional file 5: Figure S2. Phylogenetic neighbor net of global goat breeds from the two-step (excluding admixed and related animals) optimized dataset constructed with Nei’s DA distances (scale in the upper left corner) calculated with the 4-SNP blocks. [file 12864_2021_7802_MOESM5_ESM.pdf]

# Principal Component Analysis (SmartPCA) plot of *Eur2Step*

**A**

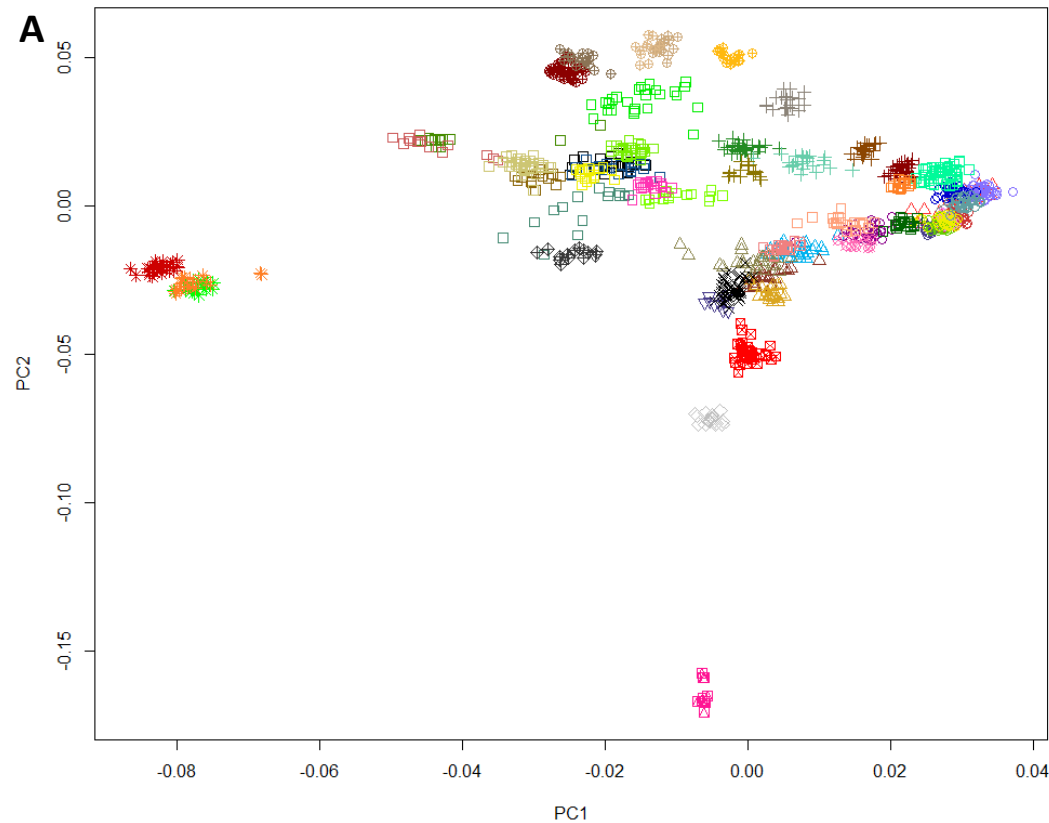

**B**

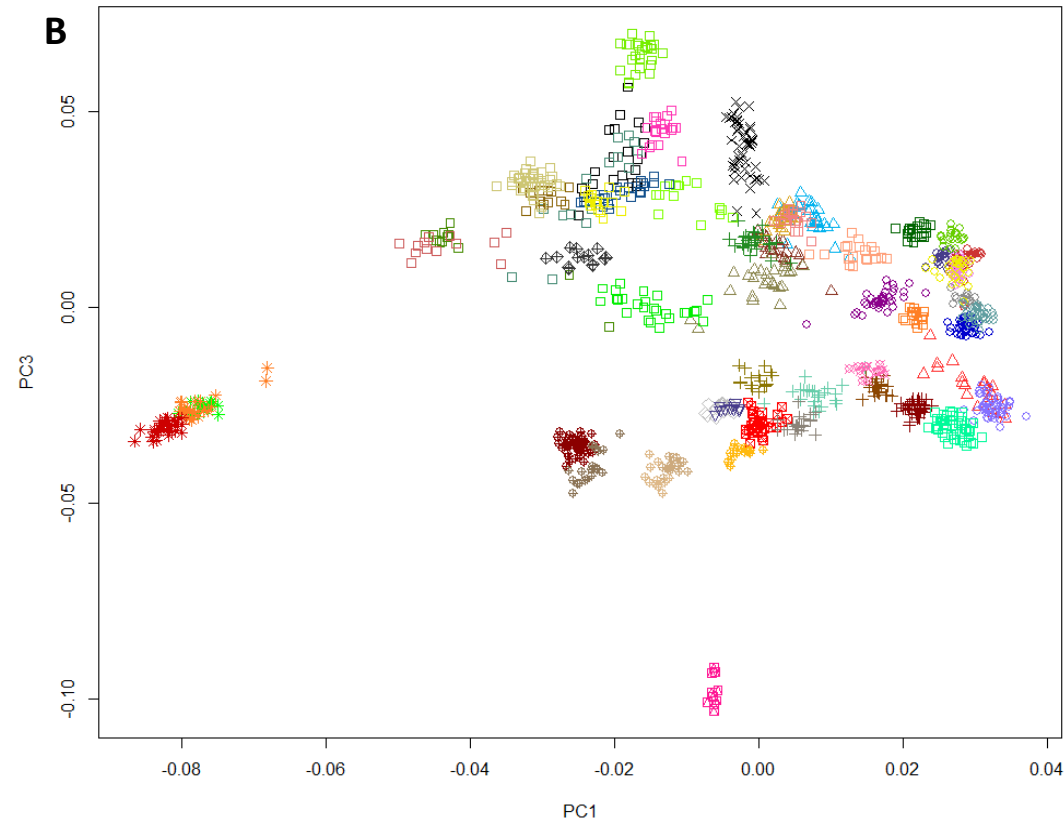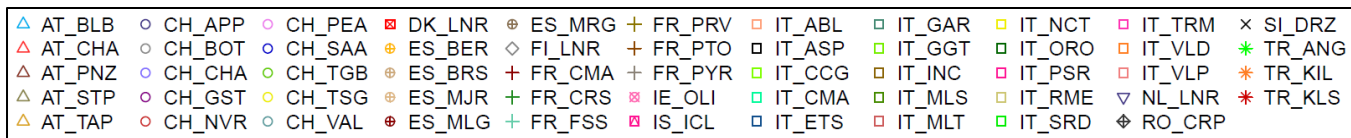

Supplement: Supplementary file 6 — Additional file 6: Figure S3. Graph of Principal Component Analysis (PCA) performed with SNP alleles of goat breeds from the Euro2Step dataset, where admixed and related animals were excluded. Besides first and second principal components (A), the third principal component was also analyzed (B). [file 12864_2021_7802_MOESM6_ESM.pdf]
